# BCL2L13 at endoplasmic reticulum-mitochondria contact sites regulates calcium homeostasis to maintain skeletal muscle function

**DOI:** 10.1016/j.isci.2024.110510

**Published:** 2024-07-14

**Authors:** Dogan Grepper, Cassandra Tabasso, Nadège Zanou, Axel K.F. Aguettaz, Mauricio Castro-Sepulveda, Dorian V. Ziegler, Sylviane Lagarrigue, Yoan Arribat, Adrien Martinotti, Ammar Ebrahimi, Jean Daraspe, Lluis Fajas, Francesca Amati

**Affiliations:** 1 Aging and Muscle Metabolism Lab, Department of Biomedical Sciences, Faculty of Biology and Medicine, University of Lausanne, Bugnon 7, Lausanne, Vaud 1005, Switzerland; 2 Institute of Sport Sciences, Faculty of Biology and Medicine, University of Lausanne, Lausanne, Vaud 1015, Switzerland; 3 Service of Endocrinology, Diabetes and Metabolism, Lausanne University Hospital and University of Lausanne, Lausanne, Vaud 1011, Switzerland; 4 Center for Integrative Genomics, Faculty of Biology and Medicine, University of Lausanne, Lausanne, Vaud 1015, Switzerland; 5 Electron Microscopy Facility, Faculty of Biology and Medicine, University of Lausanne, Lausanne, Vaud 1015, Switzerland

**Keywords:** pharmacology, cell biology

## Abstract

The physical connection between mitochondria and endoplasmic reticulum (ER) is an essential signaling hub to ensure organelle and cellular functions. In skeletal muscle, ER-mitochondria calcium (Ca^2+^) signaling is crucial to maintain cellular homeostasis during physical activity. High expression of BCL2L13, a member of the BCL-2 family, was suggested as an adaptive response in endurance-trained human subjects. In adult zebrafish, we found that the loss of Bcl2l13 impairs skeletal muscle structure and function. Ca^2+^ signaling is altered in Bcl2l13 knockout animals and mitochondrial complexes activity is decreased. Organelle fractioning in mammalian cells shows BCL2L13 at mitochondria, ER, and mitochondria-associated membranes. ER-mitochondria contact sites number is not modified by BCL2L13 modulation, but knockdown of BCL2L13 in C2C12 cells changes cytosolic Ca^2+^ release and mitochondrial Ca^2+^ uptake. This suggests that BCL2L13 interaction with mitochondria and ER, and its role in Ca^2+^ signaling, contributes to proper skeletal muscle function.

## Highlights

• Loss of Bcl2l13 negatively affects swimming capacity and skeletal muscle integrity
• Bcl2l13 loss alters mitochondrial function and morphology
• BCL2L13 localizes to ER-mitochondria contact sites
• Bcl2l13 KD increases mitochondrial Ca^2+^ uptake and regulates Ca^2+^ dynamics

## Graphical abstract

**Figure:**
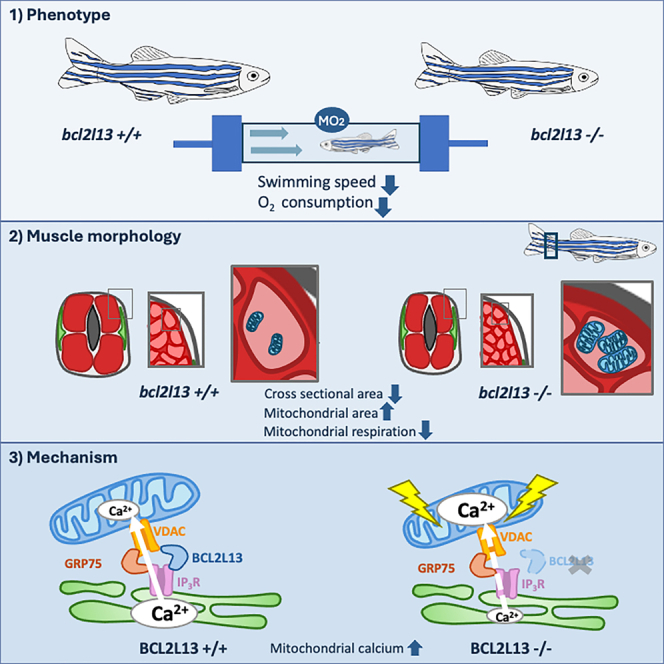

## Introduction

The transmission of calcium (Ca^2+^) from the endoplasmic or sarcoplasmic reticulum (ER/SR) to mitochondria is the key to a functional skeletal muscle. Impairments in ER/SR-mitochondria Ca^2+^ transmission are involved in reduced muscle performance during aging, exercise-induced muscle damage, and muscle disorders.^1^

Ca^2+^ transfer from the ER to mitochondria supports vital cellular mechanisms ranging from mitochondrial bioenergetics through activation of the mitochondrial dehydrogenases^2^ to reactive oxygen species (ROS) production^3^ and apoptosis.^4^ Ca^2+^ transfer takes place at specific sites of near contact between mitochondria and ER/SR.^5^ At these specific locations, called ER mitochondrial contacts (ERMCs),^6^ ER/SR Ca^2+^ release channels are physically linked through glucose-regulated protein 75 (GRP75) to the voltage-dependent anion channel (VDAC) on the mitochondrial outer membrane (MOM).^7^ The close appositions of ER/SR Ca^2+^ release channels and mitochondria favor locally high Ca^2+^ concentrations which activate the mitochondrial Ca^2+^ uniporter (MCU) and allow Ca^2+^ across the inner mitochondrial membrane (IMM).^8^^,^^9^ ER-mitochondria Ca^2+^ transmission requires strict regulation, carried out, among others, by the B cell lymphoma 2 (BCL-2) family proteins. Besides their canonical role to control MOM permeabilization,^10^ BCL-2 proteins are known to regulate apoptosis by modification of Ca^2+^ signaling at the ER and at ERMCs.^11^

BCL2L13, also known as BCL-rambo, was discovered more than two decades ago.^12^ BCL2L13 is a member of the BCL-2 family proteins and manifests all four BH domains common in BCL-2 antiapoptotic proteins. Overexpression of BCL2L13 shows apoptotic activity, which is not regulated by the interaction with other BCL-2 family proteins.^12^ Ectopic expression of BCL2L13 induces apoptosis in cellular models and in drosophila.^13^^,^^14^^,^^15^ In glioblastoma, BCL2L13 overexpression inhibits therapy-induced apoptosis.^16^ BCL2L13 has been reported to play a role in mitochondrial dynamics and mitophagy. In white adipocytes, BCL2L13 is involved in browning processes by regulating mitochondrial dynamics and biogenesis^17^ and increasing oxidative phosphorylation during adipogenesis.^18^ BCL2L13 has been compared to MOM-anchored mitophagy receptors, such as BNIP3, AMBRA1, or FUNDC1, and identified as the mammalian homolog of the yeast mitophagy receptor Atg32.^19^^,^^20^ The same authors reported that BCL2L13 induces mitophagy independently from PARKIN by recruitment of the unc-51-like kinase (ULK1) complex.^21^ Although biological properties and functions have been proposed for BCL2L13, the molecular mechanisms and its physiological and pathological roles have yet to be elucidated.^22^

We previously observed high expression of BCL2L13 in skeletal muscle of endurance-trained volunteers compared to age and gender-matched sedentary subjects.^23^ BCL2L13 was proposed as a candidate gene for adaptive evolution in the native Mexican population Raràmuri known for their high-endurance phenotype.^24^ These observations suggest a specific function of BCL2L13 in skeletal muscle of human subjects with high endurance.

Here, we generated a *bcl2l13* knockout (KO) zebrafish line with the aim to study its physiological importance. *Bcl2l13* KO fish presented reduced swimming capacity and impaired skeletal muscle structure. Skeletal muscle protein expression of targets belonging to Ca^2+^ signaling, ROS handling, and NAD+ metabolism was modified. This was accompanied by decreased mitochondrial respiration in KO fish. Investigations in cellular models revealed that BCL2L13 localizes partially to ERMCs and knocking down of BCL2l13 decreased cytosolic Ca^2+^ release and increased mitochondrial Ca^2+^ uptake. Our findings propose a novel function for BCL2L13 in Ca^2+^ regulation which could be linked to its localization at ERMCs and play an important role in skeletal muscle function.

## Results

### Loss of Bcl2l13 impairs spontaneous swimming and maximal swimming capacity in zebrafish

The zebrafish is a well-established model for studying the mechanisms that regulate skeletal muscle function.^25^ Approximately 70% of human genes have a zebrafish orthologue.^26^ Protein sequence alignment shows that BCL2L13 is highly conserved in vertebrates, from zebrafish to humans (Figure S1A).

To elucidate the physiological role of Bcl2l13, we created a zebrafish *bcl2l13* null mutant using CRISPR-Cas9 technology. DNA sequencing revealed the deletion of 5 nucleotides, resulting in a premature stop codon at the end of exon 2 (Figures S1B and S1C). Due to the lack of available antibodies for zebrafish Bcl2l13, we cloned cDNA from wild type (WT) and homozygous mutant (KO) embryos at 48 h post fertilization (hpf). Ectopic expression of FLAG-WT and FLAG-mutant *bcl2l13* (FLAG-KO) in HeLa cells confirmed that the mutation led to a loss of protein expression (Figure S1D). qPCR analysis in WT and KO fish revealed a significant decrease of *bcl2l13* mRNA levels in KO fish, suggesting that the premature stop codon increases mRNA decay (Figure S1E).

**Figure 1:**
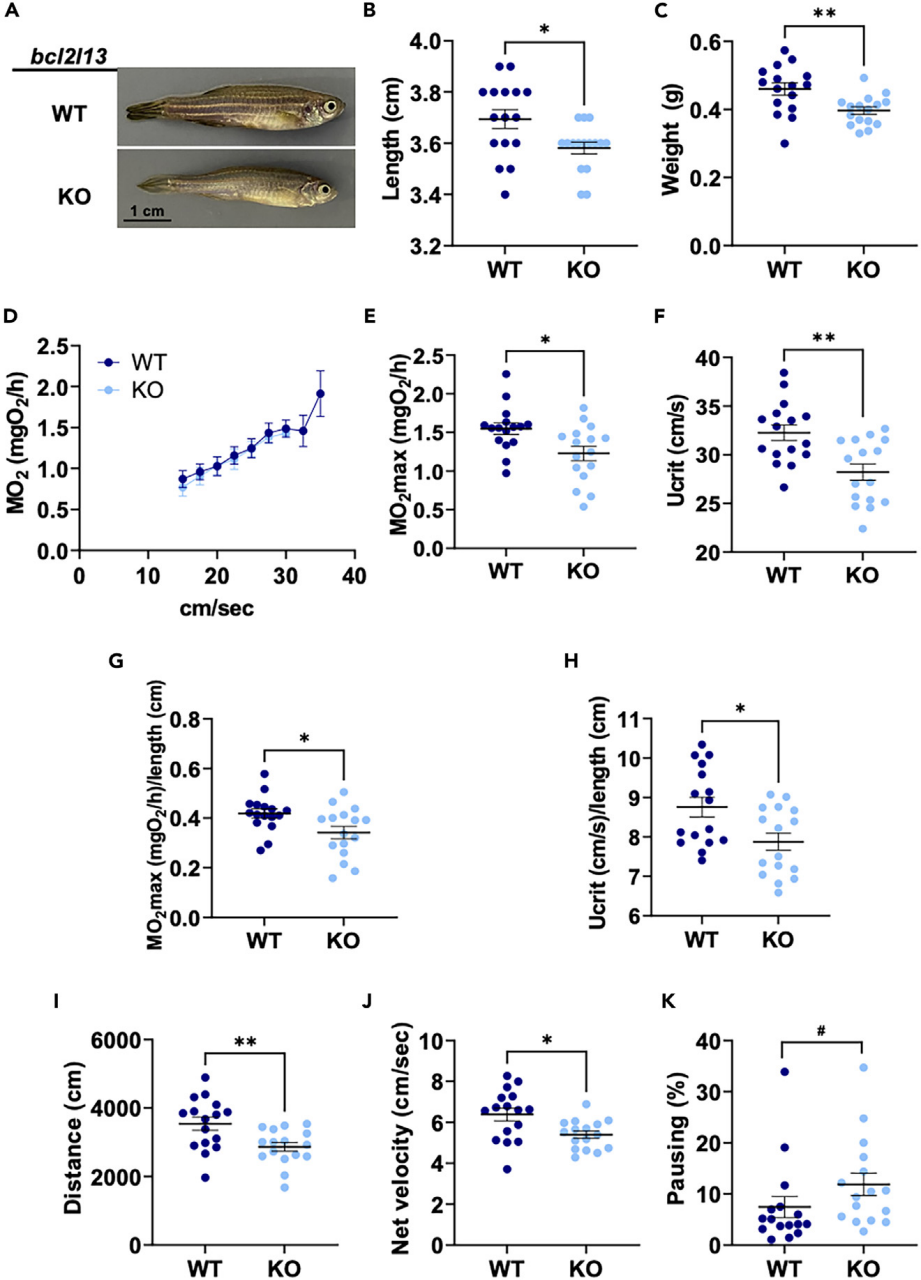
Phenotype and swimming capacity of *bcl2l13* knockout fish (A) Representative image of adult male fish. (B) Length. (C) Weight. (D) Mean MO_2_ during the incremental exercise test. (E–H) MO_2_max and Ucrit in absolute values and normalized by length. (I–K) Outcomes of the spontaneous locomotion test. For all panels, *n* = 16 fish per group from three independent cohorts. Error bars are mean ± SEM, ^∗^*p* < 0.05, ^∗∗^*p* < 0.01 (unpaired *t* test), ^#^*p* < 0.01 (Mann-Whitney). WT: wild type and KO *bcl2l13*. MO_2_ is oxygen consumption rate, Ucrit is critical swimming speed. See also Figure S1.

We compared physiological parameters in adult male WT and KO fish. Interestingly, KO fish were shorter and weighed less than WT fish (Figures 1A–1C). We performed an incremental swimming test by gradually increasing water flow rate in a hermetic chamber. We found that KO fish had a significantly lower maximal oxygen consumption rate (MO_2_max) and critical swimming speed (Ucrit) compared to WT fish (Figures 1D–1F). In fish, skeletal muscle accounts for 40–70% of body weight.^27^ Models of fish gait and speed have shown that the undulatory motion is a function of fish length rather than weight.^28^ We confirmed that the differences in MO_2_max and Ucrit remained significant even after accounting for fish length (Figures 1G and 1H). Spontaneous locomotion was decreased in KO fish compared to WT fish. KO fish traveled less with a decreased net swimming velocity and increased pausing (Figures 1I–1K). Taken together, the lower body weight and decreased swimming abilities in KO fish suggest a loss of skeletal muscle.

### Skeletal muscle fibers are affected in *bcl2l13* KO fish

**Figure 2:**
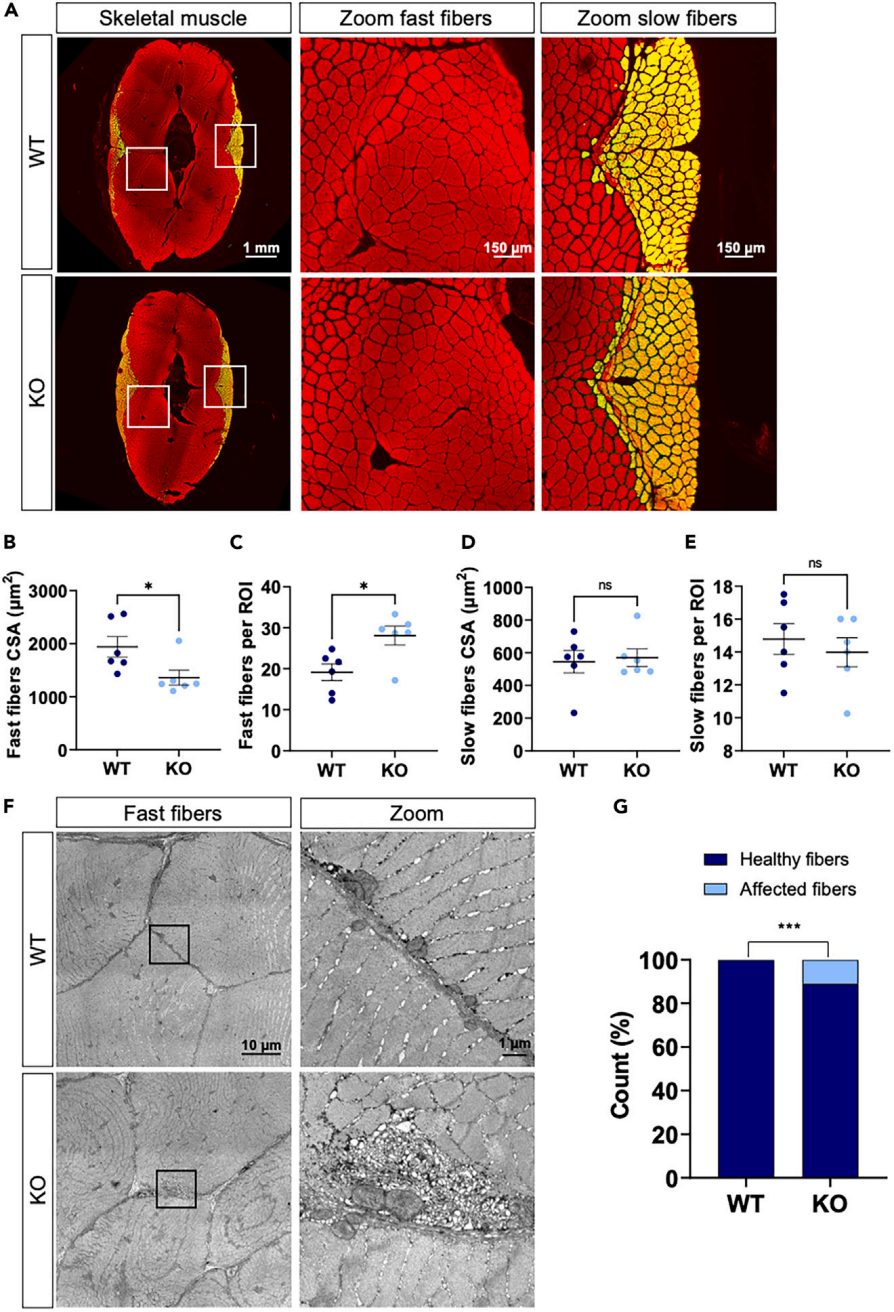
Skeletal muscle structure of WT and KO fish (A) Representative immunohistochemistry of transverse muscle sections of WT and KO fish stained with phalloidin (red). Slow fibers are marked with an antibody against MYH7B (green, merge is yellow). (B) Mean CSA of fast muscle fibers per fish. (C) Mean number of fast fibers per ROI per fish. (D) Mean CSA of slow muscle fibers per fish. (E) Mean number of slow fibers per ROI per fish. (F) Representative electron micrographs of fast muscle fibers of WT and KO fish. (G) Contingency graph for healthy fibers vs. fibers with SR accumulation. For (C–E) *n* = 6 fish per group. Error bars are mean ± SEM, ^∗^*p* < 0.05 (unpaired *t* test). For (G) *n* = 8 fish per group. *p* = 0.0007 (Fisher’s exact test). Squares (white in A, black in F) represent the regions that are zoomed out. WT: wild type and KO: Mutant *bcl2l13*. CSA is cross-sectional area; ROI is region of interest.

To investigate the impact of the loss of Bcl2l13 on skeletal muscle, we analyzed the cross-sectional area (CSA) of fast and slow twitch muscle fibers. CSA is closely related to skeletal muscle health and is an indicator for muscle atrophy.^29^ Fast twitch muscle fibers were significantly smaller in KO zebrafish compared to WT animals. This was indicated by the decreased mean CSA and the increased number of fibers in the surface analyzed (Figures 2A–2C). No changes were observed in slow twitch muscle fibers (Figures 2D and 2E).

We conducted a qualitative analysis of electron microscopy micrographs in fast twitch muscle fibers of KO and WT fish, which revealed the presence of tubular aggregates in the subsarcolemmal region of a subset of KO fibers (Figure 2F). These tubular aggregates were observed in 11% of the KO fibers but not in any of the WT fibers (Figure 2G). Tubular aggregates originate from SR membranes and are caused by altered Ca^2+^ homeostasis.^30^^,^^31^ Although observed in various neuromuscular diseases, this phenotype is typically seen in myopathies leading to muscle wasting.^32^^,^^33^ Overall, our results indicate that the loss of Bcl2l13 has a detrimental impact on skeletal muscle structure, potentially altering Ca^2+^ homeostasis.

### Untargeted proteomics reveal changes in skeletal muscle Ca^2+^ signaling

**Figure 3:**
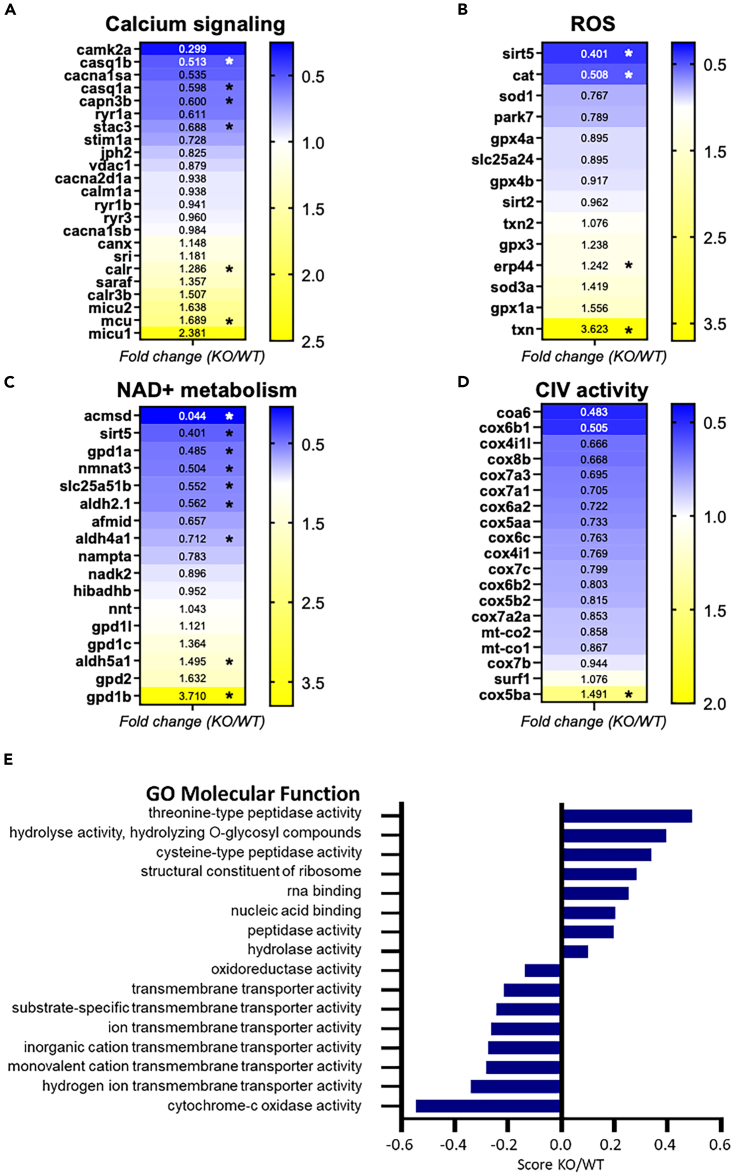
Skeletal muscle proteomics of KO and WT zebrafish (A–D) Fold change heat maps in selected proteins associated with different cellular functions. (E) Enrichment score KO/WT of significantly modified protein groups related to gene ontology molecular function (GOMF). The score is derived from 1D annotation enrichment analysis.^40^ For all panels, *n* = 8 fish per group. ^∗^*p* < 0.05 (*t* test with multiple testing correction: permutation-based FDR at 0.05). See also Figures S2 and S4, and Tables S2 and S3.

After centrifugation-based fractionation to reduce myofibrillar proteins, the proteomes of skeletal muscle lysates from WT and KO fish were compared. The proteomics analysis confirmed the absence of Bcl2l13 in KO fish (Table S2). In one fish, a single low-intensity peptide was detected for Bcl2l13 that was considered an artifact. The loss of Bcl2l13 resulted in differential modulation of targets related to Ca^2+^ signaling, ROS handling, and NAD+ transport, consumption, and utilization (Figures 3A–3C and Table S3), as well as electron transport chain (ETC) complex IV (CIV) subunits (Figure 3D). Gene ontology (GO) enrichment analyses of molecular function revealed decreased expression of transmembrane transport and cytochrome *c* oxidase activity proteins in KO fish compared to WT (Figure 3E). These results suggest that skeletal muscle Ca^2+^ homeostasis is altered in *bcl2l13* KO fish, affecting mitochondrial protein expression and ROS production. This causality is well described in several studies using zebrafish and mouse mutant models for Ca^2+^ signaling proteins suggesting that Ca^2+^ dysregulation induces mitochondrial damage and ROS production leading to muscle dysfunction.^34^^,^^35^^,^^36^^,^^37^^,^^38^^,^^39^

Furthermore, GO enrichment analyses revealed that both protein degradation and protein synthesis are upregulated. Indeed, increased threonine- and cysteine-type peptidase activity, and hydrolase activity in KO fish, are processes involved in protein degradation, while increased structural constituent of ribosome, RNA binding, and nucleic acid binding in KO fish suggest higher protein synthesis (Figures 3E and S2). The most upregulated protein groups suggest that protein breakdown could be faster than protein synthesis in KO fish. The regulation of skeletal muscle mass strongly depends on protein turnover. Muscle atrophy occurs when protein degradation pathways are hyperactivated and exceed protein synthesis rates, leading to skeletal muscle loss.^41^ This finding aligns with the observed decrease in CSA of fast muscle fibers in KO fish.

### Mitochondrial content, morphology, and respiration in *bcl2l13* KO muscle

**Figure 4:**
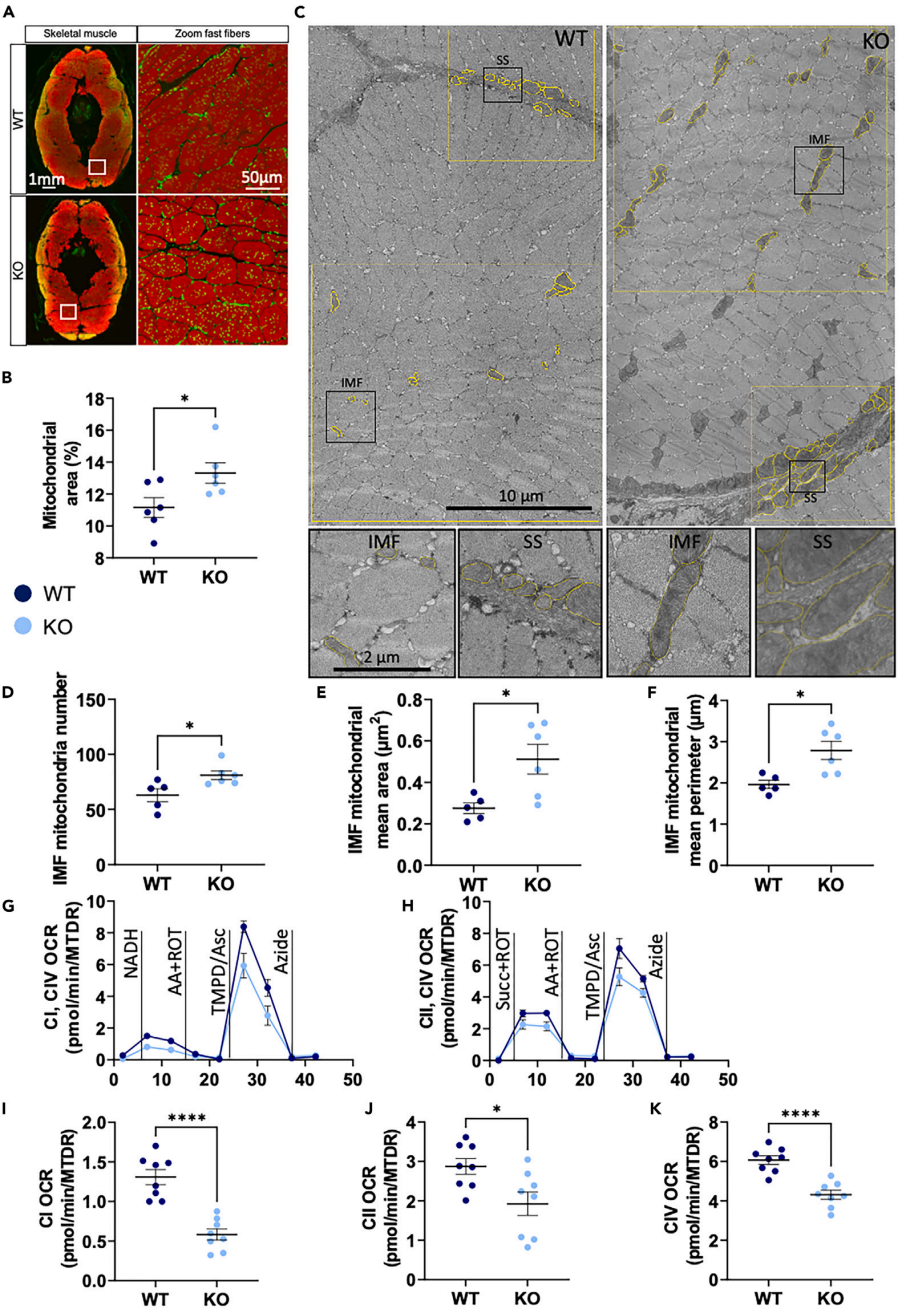
Mitochondrial content, morphology and respiration in *bcl2l13* KO muscle (A) Representative immunohistochemistry of transverse muscle sections of WT and KO fish stained with phalloidin (red). Mitochondria are marked with an antibody against MCTO1 (green, merge is yellow). (B) Mitochondrial area in fast muscle fibers. (C) Representative micrographs with ROI and mitochondria circling (yellow overlays) and zoomed section (black squares). (D–F) Analyses of IMF mitochondrial parameters. (G–K) Oxygen consumption rates normalized to mitochondrial content with Mitotracker Deep Red (OCR/MTDR). Black vertical lines indicate addition of substrates and electron transport chain (ETC) complexes inhibitors. (H–J) Mean ETC complex I activity (CI), complex II activity (CII), and complex IV (CIV) activity. For (D–F), *n* = 5 WT and 6 *bcl2l13* KO zebrafish. For (G–K), *n* = 8 biological replicates per group. For all panels, error bars are mean ± SEM. ^∗^*p* < 0.05, ^∗∗∗∗^*p* < 0.0001 (unpaired *t* test). WT: Wild type and KO: Mutant *bcl2l13*. IMF is intermyofibrillar, SS is subsarcolemmal, CI–CIV are ETC complexes I to IV, NADH is beta-nicotinamide adenine dinucleotide, Succ is succinate, ROT is rotenone, AA is antimycin A, TMPD is N,N,N′,N′-tetramethyl-1,4-phenylendiamin, Asc is ascorbic acid. See also Figure S3.

Our proteomic analysis revealed changes primally in processes associated with mitochondria function and Ca^2+^ homeostasis both of which can impact mitochondrial morphology and respiration.^42^^,^^43^ We measured mitochondrial area in fast twitch fiber muscle sections and found increased mitochondrial area in KO fish compared to WT (Figures 4A and 4B). Using electron microscopy micrographs, we analyzed the surface area and morphology of intermyofibrillar (IMF) and subsarcolemmal (SS) mitochondria (Figure 4C). In the IMF region, mitochondria were more abundant in KO animals compared to WT (Figure 4D). The morphological analysis showed that IMF mitochondria in KO fish were larger (Figure 4E) without changes in shape; they had an increased perimeter (Figure 4F), Feret, and min Feret (Figures S3A andS3B) while all shape descriptors (circularity, roundness, and aspect ratio) were not significantly different (Figures S3C–S3E). In the SS region, mean mitochondria area was the only parameter that tended toward significance (Figures S3F–S3M). Overall, the morphological analysis shows that IMF mitochondria in KO fish are more abundant and larger but not more elongated than WT fish. Larger mitochondria have been shown to exhibit faster and higher Ca^2+^ uptake.^42^ Excessive Ca^2+^ uptake leads to mitochondrial dysfunction and swelling caused by mitochondrial permeability transition which could explain the appearance of enlarged mitochondria.^44^

Although no changes were observed in our proteomics analysis, we analyzed transcript levels of genes linked to mitophagy and mitochondrial biogenesis, which could contribute to changes of the mitochondrial network. None of the investigated targets showed differences between KO and WT fish (Figures S4A–S4G).

To investigate the impact on mitochondrial respiration, we measured the activities of ETC complexes I, II, and IV in KO and WT fish skeletal muscle (Figures 4G–4K). We found that the activities of all three complexes measured were decreased in KO fish, with a strong reduction in complex I and IV activity. This finding confirms that altered protein expression and changes in mitochondrial morphology led to decreased mitochondrial function in skeletal muscle of *bcl2l13* KO fish.

### Ca^2+^ homeostasis pathways are compromised KO fish

In KO fish skeletal muscle, mRNA levels of *mcu*, which is responsible for mitochondrial Ca^2+^ uptake tended toward a significant increase (Figure S4H). SR Ca^2+^ handling genes *casq1a* and *casq1b* were increased (Figures S4I and S4J). Interestingly, *casq1* mRNA levels were increased, whereas Casq1 protein levels were decreased in *bcl2l13* KO fish. This suggests a high turnover of Casq1. In a study on dogs with tachypacing-induced heart failure, high CASQ2 turnover was linked to increased muscle damage.^45^

The pyruvate dehydrogenase (PDH) complex converts pyruvate to acetyl-CoA, which is crucial for mitochondrial metabolism. The pyruvate dehydrogenase kinase (PDK) phosphorylates the PDHE1α subunit at three sites, which inactivates the complex.^46^ On the other hand, high mitochondrial Ca^2+^ activates PDH phosphatase, which dephosphorylates PDHE1α, leading to activation of the PDH complex.^47^ We found that Pdh1α was less phosphorylated on Serine^293^ (S^293^) in skeletal muscle of KO fish suggesting higher mitochondrial Ca^2+^ uptake (Figures S4K and S4L). Our results show that mitochondrial respiration is decreased in KO fish skeletal muscle, although dephosphorylated Pdh1α would suggest the opposite. We postulate that this discrepancy is due to mitochondria in a state of Ca^2+^ overload. While low amounts of Ca^2+^ are necessary for oxidative phosphorylation (OXPHOS), high mitochondrial Ca^2+^ impairs mitochondrial energy production and contributes to long-term organ dysfunction.^48^

### BCL2L13 is at ERMCs but does not affect EMRCs size or number

**Figure 5:**
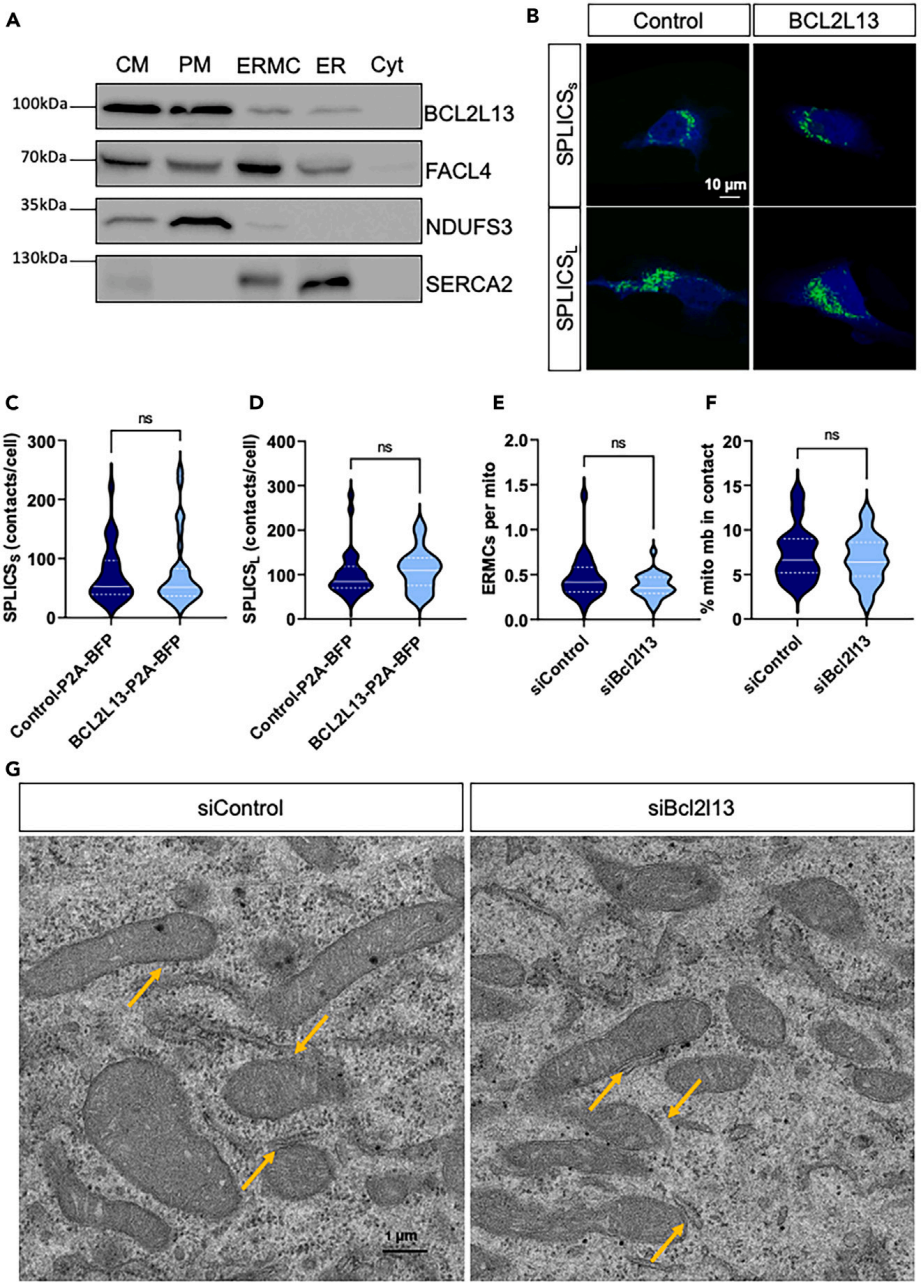
Subcellular localization of BCL2L13 and ERMCs quantifications (A) Representative western blot of isolated subcellular fractions. Expression of BCL2L13, FACL4, NDUFS3, and SERCA2 in crude mitochondria (CM), pure mitochondria (PM), EMRCs, ER, and cytosol (Cyt). Experiments were repeated 4 times. (B) Representative confocal microscopy images of HeLa cells expressing SPLICS_S/L_ probes (green) and BFP from P2A constructs (blue). (C) Quantification of SPLICS_S_ and (D) SPLICS_L_ contacts in HeLa cells expressing control-P2A-BFP (Control) or BCL2L13-P2A-BFP (BCL2L13). *n* = 30 cells from four independent transfections. (E) Number of ERMCs per mitochondria and (F) percentage of the mitochondrial membrane in contact with ERMCs (% mito mb in contact) measured in electron micrographs from siControl and siBCL2L13 transfected C2C12 myotubes. (G) Representative electron micrographs of C2C12 myotubes in siControl and siBCL2L13 transfected cells. Orange arrows indicate examples of ERMCs. *n* = 27 micrographs (4.95 × 4.95 μm) per condition from four independent experiments. For all panels, the median is shown by a white full line while quartiles are dotted lines. See also Figures S5 and S6.

Our results suggest that BCL2L13 regulates mitochondrial function and skeletal muscle health by controlling Ca^2+^ homeostasis. The main sites of SR-mitochondria transition are ERMCs.^6^ Although previous studies have explored BCL2L3 functions that could be linked to ERMCs,^16^^,^^49^^,^^50^ the subcellular localization of BCL2L13 has not been yet examined. We investigated the subcellular localization of BCL2L13 in human myoblasts (HIM) using subcellular fractionation. HIM cells serve as a valuable model to demonstrate the relevance of our findings in a human muscle model. In addition to its typical mitochondrial localization, we identified BCL2L13 at ERMCs and ER (Figure 5A). The localization of BCL2L13 at ERMCs may play a crucial role in Ca^2+^ signaling. Our discovery of BCL2L13 localization at ERMCs is supported by ERMCs proteomics studies in HEK cells, human fibroblasts, mouse brain, and more recently in rabbit heart and skeletal muscle.^23^^,^^51^^,^^52^^,^^53^^,^^54^^,^^55^^,^^56^

Changes in ERMCs tethering are known to directly impact Ca^2+^ transfer between the two organelles.^8^^,^^57^ Using split-GFP-based contact site sensors to investigate the potential role of BCL2L13 in stabilizing EMRCs, we measured short range interactions (≈8–10 nm, SPLICS_S_) and long range interactions (≈40–50 nm, SPLICS_L_) between mitochondria and ER. SPLICS_S_ and SPLICS_L_ detected contact sites showed no alterations in HeLa cells expressing BCL2L13-P2A-BFP compared to control-P2A-BFP (Figures 5B–5D).

After validating the silencing of Bcl2l13 in C2C12 cells (Figures S5A and S5B), we quantified ERMCs and mitochondria in electron micrographs. While we observed a higher number of mitochondria in Bcl2l13 knockdown cells, the number of ERMCs per cell, contacts per mitochondria and percentage of the mitochondrial membrane for each mitochondrion in contact with the ER were not different between siBcl2l13 and siControl (Figures 5E–5G, S6A, and S6B). The minimal and maximal distance between ER and mitochondria were not affected by knocking down Bcl2l13 (Figures S6C and S6D). The mean mitochondrial circumference, total mitochondrial length, and the total length of mitochondria in contact with the ER remained unchanged in siBcl2l13 cells compared to siControl cells (Figure S6). Our results indicate that BCL2L13 does not directly affect ERMCs tethering and the mitochondrial network is not influenced by short-term depletion of BCL2L13. A direct impact of BCL2L13 on ER-mitochondria Ca^2+^ transfer could explain a delay between the loss of BCL2L13 and the onset of mitochondrial damage, where sustained stress from excessive mitochondrial Ca^2+^ uptake leads to mitochondrial dysfunction.

### BCL2L13 regulates Ca^2+^ flux in C2C12 myotubes

To understand the impact of BCL2L13 on Ca^2+^ signaling, we measured the concentration of Ca^2+^ signaling proteins and the phosphorylation state of PDH1αS^293^ in Bcl2l13 knockdown C2C12 myotubes compared to controls (Figures S5C–S5F). The lack of differences observed suggests that short-term depletion of BCL2L13 is not enough to induce a transcriptional adaptation in Ca^2+^ signaling proteins.

**Figure 6:**
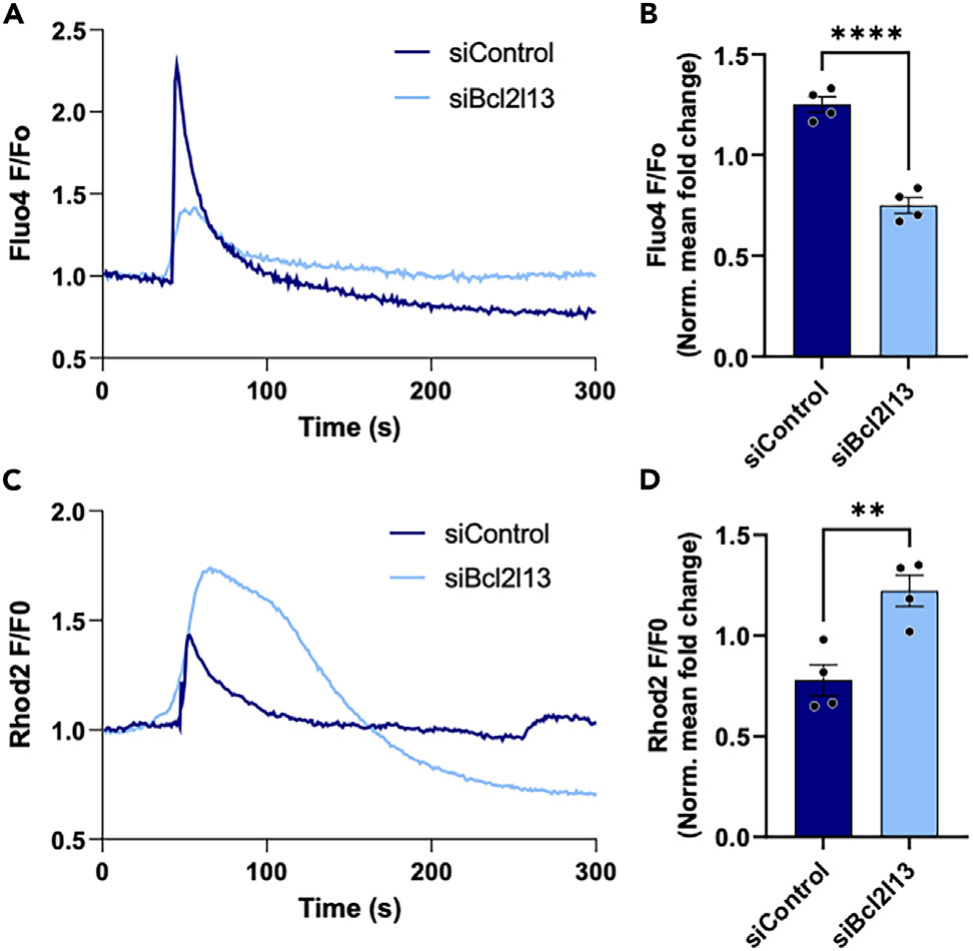
Ca^2+^ dynamics in siBCL2L13 and siControl C2C12 myotubes (A) 300 s confocal time-lapse recording of cytosolic Ca^2+^ levels. (B) Quantification of normalized Fluo-4 fluorescence in response to 2.5 mM caffeine. (C) 300 s confocal time-lapse recording of mitochondrial Ca^2+^ levels. (D) Normalized Rhod-2 fluorescence in response to 1 μM thapsigargin. (A–D) *n* = 4 independent transfections per condition. (B and D) peak fluorescence was normalized to baseline. Error bars are mean ± SEM, ^∗^*p* < 0.05, ^∗∗^*p* < 0.01, ^∗∗∗∗^*p* < 0.0001 (unpaired *t* test). See also Figure S5.

To further investigate the effects of the depletion of BCL2L13 on Ca^2+^ dynamics, we measured the amount of Ca^2+^ stored in the SR of Bcl2l13 knockdown and control cells. By treating the cells with caffeine, which triggers SR Ca^2+^ release through its agonistic effect on RyR1,^43^ we found that the amount of Ca^2+^ released from the SR in response to caffeine was lower in Bcl2l13 knockdown myotubes, indicating reduced Ca^2+^ stores (Figures 6A and 6B). Consistent with our findings, other members of the BCL-2 protein family have been shown to influence RyR-mediated Ca^2+^ release.^58^^,^^59^

Furthermore, we wanted to investigate if BCL2L13 directly impacts mitochondrial Ca^2+^ uptake. To avoid bias of SR Ca^2+^ reuptake through sarcoplasmic/endoplasmic Ca^2+^ ATPase (SERCA), we used thapsigargin (TG) to deplete SR Ca^2+^ stores. We found that mitochondrial Ca^2+^ uptake was increased in Bcl2l13 knock down cells in response to TG (Figures 6C and 6D). Our results suggest increased mitochondrial Ca^2+^ uptake in the absence of BCL2L13.

Mitochondrial Ca^2+^ overload is detrimental, as it affects the mitochondrial membrane potential and leads to mitochondrial swelling and apoptosis. BCL-2 family proteins are known to modulate mitochondrial Ca^2+^ entry to regulate cell survival.^60^^,^^61^ We found that prolonged exposure to TG leads to increased CASP3 cleavage in siBcl2l13 myotubes but not in siControl (Figures S5E and S5F), suggesting that BCL2L13 protects mitochondria from apoptotic changes during periods of stress.

## Discussion

As recently expressed by Kataoka,^22^ who discovered BCL2L13 two decades ago,^12^ the molecular function, physiological, and pathological roles of BCL2L13 have not yet been elucidated.^22^^,^^62^ Our study adds a novel perspective to answer these questions by focusing on skeletal muscle and the role of BCL2L13 in Ca^2+^ signaling.

The loss of *bcl2l13* affected physiological parameters in adult fish including decreased spontaneous swimming and maximal swimming capacity. In zebrafish, decreased Ucrit and MO_2_ have been previously linked to mitochondrial dysfunction and skeletal muscle atrophy.^63^ Similarly to old zebrafish,^55^^,^^64^ who present decreased swimming capacity, zebrafish lacking *bcl2l13* show signs of muscle loss with decreases in fast fiber CSA. In humans, increased BCL2L13 expression in skeletal muscle was found in endurance-trained subjects and the native Mexican population Rarámuri known for their high-endurance phenotype.^23^^,^^24^ Based on these findings, we hypothesize that the decreased swimming capacity in *bcl2l13* KO fish is a consequence of skeletal muscle dysfunction. This notion is supported by our finding that the loss of *bcl2l13* reduces fiber size and provokes abnormal SR accumulation in fast fibers resembling the phenotype of tubular aggregate myopathy linked to mutations in proteins involved in store operated Ca^2+^ entry such as STIM1, ORAI1, and CASQ1.^65^

It is well established that Ca^2+^ handling abnormalities are involved in muscle dysfunction and myopathies.^66^^,^^67^ Interestingly, the skeletal muscle of KO fish presented several modified proteins involved in Ca^2+^ signaling, such as Stac3, known to be mutated in a Native American myopathy.^68^ Stac3 loss of function zebrafish mutants (*Stac3*^*mi34*^) exhibited defective swimming and locomotor dysfunction at 48 hpf.^68^ Furthermore, *bcl2l13* KO fish had increased Mcu expression and decreased Casq1a, Casq1b, Ryr1a, and calpain-3 (Capn3b) protein levels. These modifications mirror altered protein expression observed in muscle biopsies from patients with sporadic inclusion body myositis.^69^ Calpain-3 loss of function mutations cause limb-girdle muscular dystrophy type2a^70^ and SR Ca^2+^ release is altered in skeletal muscle from calpain-3 deficient mice.^71^ In light of these previous observations, our findings in *bcl2l13* KO fish support the hypothesis that the observed muscle dysfunction is due to altered Ca^2+^ homeostasis.

Studies in *ryr1b* loss of function zebrafish mutants proposed that reduced skeletal muscle Ca^2+^ transients lead to altered oxidoreductase activity, nicotinamide and NADP metabolic processes, and increased mitochondrial ROS production.^34^^,^^35^ Comparably, our KO fish exhibit significant changes in ROS handling proteins and NAD+ metabolism, as well as decreased oxidoreductase activity. Studies in CASQ1-null mice and leaky RYR1 mutant models indicate that Ca^2+^ dysregulation induces mitochondrial damage and ROS production, ultimately leading to muscle dysfunction.^36^^,^^37^^,^^38^^,^^39^ These studies suggest a strong link between skeletal muscle Ca^2+^ dyshomeostasis and impaired mitochondrial function. Our results in *bcl2l13* KO fish showing Pdh1α dephosphorylation concurrent with increases in *mcu* mRNA and protein levels, suggest an increased mitochondrial Ca^2+^ uptake in KO fish.^47^^,^^72^ Despite that PDHE1α phosphorylation state favors increased OXPHOS, we observed decreases in mitochondrial respiration in the skeletal muscle of KO fish, in conjunction with modified expression of proteins in NAD+ metabolism and ROS handling, and decreased expression of CIV subunits. Given that high mitochondrial Ca^2+^ impairs mitochondrial function,^48^ we propose that the imbalance of Ca^2+^ homeostasis in *bcl2l13* KO fish is the driver of this mitochondrial dysfunction.

We discovered that BCL2L13 partially resides at ERMCs and focused on its involvement in Ca^2+^ signaling. It is important to acknowledge that we did not focus on the most studied function of BCL2L13 in mitophagy,^20^^,^^21^^,^^73^^,^^74^^,^^75^ although it may be linked to ERMCs, altered protein turnover, and mitochondrial quality as observed in our experiments. Indeed, in addition to Ca^2+^ signaling, ERMCs serve as crucial signaling hubs for various processes such as lipid trafficking, mitochondrial dynamics, and autophagosome formation. Many of these processes involve BCL2L13. For example, it is known that ERMCs play a role in ceramide transport and synthesis^76^ and BCL2L13 has been found to bind ceramide synthases 2 and 6, leading to the inhibition of therapy-induced apoptosis in glioblastoma.^16^ During mitochondrial fission, ER tubules wrap around mitochondria and recruit dynamin-related protein 1 (DRP1) to ERMCs to complete the fission process.^77^ BCL2L13 has been found to induce mitochondrial fission independently of DRP1, although it is still unclear if mitochondrial fission induced by BCL2L13 requires contact between mitochondria and ER.^20^ Autophagosomes form at ERMCs^78^ with the contribution of ARL6IP1. ARL6IP1, which interacts with BCL2L13 to regulate mitophagy,^49^ is known to localize at ERMCs and aid in autophagosome formation by expanding the phagophore from the ER. Additionally, ARL6IP1 is implicated in hereditary spastic paraplegia.

Our findings are consistent with other studies that explored the role of BCL-2 family proteins. It is known that several members of the BCL-2 family are located at ERMCs and control intracellular Ca^2+^ signaling.^79^^,^^80^ These proteins play a crucial role in fine-tuning Ca^2+^ signaling by interacting with Ca^2+^ release and uptake channels at ERMCs, ultimately regulating mitochondrial bioenergetics and cell survival.^11^ For BCL-2 and BCL-xL, which, similarly to BCL2L13, express all four BH domains, it has been shown that the N-terminal BH4 domain is crucial for the interaction with VDACs, RyRs, and IP3Rs.^58^^,^^59^^,^^81^^,^^82^^,^^83^^,^^84^ Bcl-wav, a BCL-2 related multidomain apoptosis accelerator found in fish and frogs, was shown to act on intracellular Ca^2+^ trafficking through its interaction with Vdac1 and Mcu in zebrafish early development.^85^ In two different studies using human cultured cells, BCL2L13 has been shown to interact with VDAC1 and GRP75 to promote the activation of effector caspases.^14^^,^^50^ We propose that the interaction of BCL2L13 with Ca^2+^ signaling channels such as VDAC1 could be crucial in modifying Ca^2+^ dynamics, especially mitochondrial Ca^2+^ uptake. Further studies are required to elucidate the exact mechanism of BCL2L13 in Ca^2+^ signaling and how this function is related to its subcellular localization and molecular interactors.

In conclusion, the loss of *bcl2l13* in zebrafish reduces swimming capacities and maximal oxygen consumption rate. The fast-twitch skeletal muscle of KO fish showed decreased fiber size, tubular aggregates, mitochondrial dysfunction, and changes in mitochondrial morphology. Proteomics analysis revealed that Ca^2+^ signaling proteins were differentially expressed in KO and WT fish muscle. We found that BCL2L13 localizes at ERMCs and affects Ca^2+^ dynamics in mammalian cells. In summary, we demonstrate that the loss of BCL2L13 impacts muscle function and quality through its effect on ER and mitochondrial calcium flux. We propose a novel role of BCL2L13 in the regulation of Ca^2+^ homeostasis to preserve skeletal muscle function.

### Limitations of the study

The zebrafish is a well-recognized model to study skeletal muscle, but it is currently limited in terms of specific antibodies available. For this reason, we were not able to use BCL2L13 antibodies to study interactors of this protein in zebrafish. Although we used multiple models to validate the role of BCL2L13, the evidence connecting Ca^2+^ signaling disruptions to muscle defects *in vivo* is indirect. The use of multiple models allowed to obtain a constellation of results that point to its function without the full mechanism of action identified. Each model has been used with a clear rationale. Human myoblasts were used to analyze the subcellular localization of BCL2L13 to show the relevance of our observations for previous studies conducted in humans. We chose C2C12 to measure SR Ca^2+^ release and mitochondrial Ca^2+^ uptake because these cells are known to be a good model for Ca^2+^ signaling and their contractile capacity in the dish, which is not the case of human primary myotubes. HeLa cells have the technical advantage of being easy to transfect compared to C2C12 cells or human myoblasts. We used HeLa for the SPLICs experiments to ensure a satisfactory transfection rate and acquire enough images for statistical analysis. Table S4 presents all purposes and rationale for each model used. Further *in vivo* studies building upon these observations will be needed for experimental validation.

## STAR★Methods

### Key resources table

**Table:** 

REAGENT or RESOURCE	SOURCE	IDENTIFIER
**Antibodies**		

anti-myosin heavy chain MYH7B	Developmental Studies Hybridoma Bank, University of Iowa, Iowa City, IA, United States	Cat# S58; RRID: AB_528377
goat anti-mouse IgG-FITC	Santa Cruz Biotechnology	Cat# SC-2010; RRID: AB_631735
mouse anti-MYH2	Santa Cruz Biotechnology	Cat# A4.74; RRID: AB_528383
mouse anti-ATP5a	Abcam	Cat# ab14748; RRID: AB_301447
mouse anti-PDHα	Abcam	Cat# ab110333; RRID: AB_10862029
rabbit anti-PDH E1α phospho-serine 293	Abcam	Cat# ab177461; RRID: AB_2756339
rabbit anti-BCL2L13 (N-ter)	Sigma	Cat# HPA050377; RRID: AB_2681107
rabbit anti-BCL2L13 (center)	Sigma	Cat# HPA030994; RRID: AB_10795024
mouse anti-FACL4	Abcam	Cat# ab155282; RRID: AB_2714020
mouse anti-NDUFS3	Abcam	Cat# ab14711; RRID: AB_301429
mouse anti-SERCA2	Abcam	Cat# ab150435; RRID: AB_2910256
rabbit anti-RyR1	Marks’ lab, Columbia University, NY, USA	Cat# 5,029, Aa 1327–1339; RRID: N/A
rabbit anti-MCU	Sigma	Cat# HPA016480; RRID: AB_2071893
mouse anti-SERCA1 ATPase	Abcam	Cat# ab2819; RRID: AB_2061279
mouse anti-αTubulin	Sigma	Cat# T6199; RRID: AB_477583
mouse anti-IP3R-I/II/III	Santa Cruz Biotechnology	Cat# sc-377518; RRID: AB_2637028
rabbit anti-cleaved Caspase 3	Cell Signaling Technology	Cat# 9661; RRID: AB_2341188
HRP-linked secondary antibodies anti-mouse IgG	Cell Signaling Technology	Cat# 7076; RRID: AB_330924
HRP-linked secondary antibodies anti-rabbit IgG	Cell Signaling Technology	Cat# 7074; RRID: AB_2099233

**Bacterial and virus strains**		

Subcloning Efficiency™ DH5α™ Competent Cells	Invitrogen, ThermoFischer Scientific	18265017

**Chemicals, peptides, and recombinant proteins**		

Truecut™ Cas9 Protein v2	Invitrogen, ThermoFischer Scientific	A36498
Invitrogen™ Gateway™ BP Clonase™ II Enzyme mix	Invitrogen, ThermoFischer Scientific	11789020
Gateway™ LR Clonase™ II Enzyme mix	Invitrogen, ThermoFischer Scientific	11791020
Rhod-2, AM, cell permeant	Invitrogen, ThermoFischer Scientific	R1244
Fluo-4, AM, cell permeant	Invitrogen, ThermoFischer Scientific	F14217
Phalloidin CruzFluor^TM^ 647 Conjugate	Santa Cruz Biotechnology	sc-363797
Hoechst Nucleic Acid Stain	Invitrogen, ThermoFischer Scientific	H3570
MitoTracker™ Dyes for Mitochondria Labeling	ThermoFischer Scientific	M22426

**Critical commercial assays**		

Gene Art precision gRNA synthesis kit	Invitrogen, ThermoFischer Scientific	A29377
MinElute Gel Extraction kit	Qiagen	28604
Power SYBR Green PCR Master Mix	Thermo Fisher Scientific	4367659
GoScript Reverse Transcription Mix, Random Primers	Promega	A2801
Pierce MemCode™ Reversible Protein Stain Kits for Western Blotting	Thermo Fisher Scientific	24585
MISSION® esiRNA targeting mouse Bcl2l13	Sigma-Aldrich	EMU065241
MISSION® esiRNA targeting FLUC	Sigma-Aldrich	EHUFLUC

**Deposited data**		

Zebra fish (*Danio rerio*) reference proteome based on the UniProt database	www.uniprot.org	Version of June 26^th^, 2020, containing 46’847 sequences
All raw MS data together with raw output tables	ProteomeXchange^86^^,^^87^ data repository (www.proteomexchange.org)	PXD044765

**Experimental models: Cell lines**		

Human, HeLa, human epitheloid cervix carcinoma	Sigma-Aldrich	93021013
Human, human immortalized myoblasts	Laboratory of Francesca Amati	N/A
Mouse, C2C12, mouse C3H muscle myoblast	Sigma-Aldrich	91031101

**Experimental models: Organisms/strains**		

Zebrafish *(Danio rerio)*, Oregon AB	Laboratory of Francesca Amati	N/A
Zebrafish (Danio rerio), *bcl2l13-/-*	This study	ZFIN.org, line designation: ula2

**Oligonucleotides**		

Primers for genotyping, cloning and qPCR see Table S1	This paper	N/A

**Recombinant DNA**		

Plasmid, pCSDest2	Villefranc et al.^88^	RRID:Addgene_22424
Plasmid, pDonR2R3-p2a-BFP	This paper	N/A
Plasmid, pCI-CMV-3XflagN	Arribat et al.^89^	N/A
Plasmid, SPLICS_S_-P2A^ER-MT^	Vallese et al.^90^	RRID:Addgene_164108
Plasmid, SPLICS_L_-P2A-^ER-MT^	Vallese et al.^90^	RRID:Addgene_164107

**Software and algorithms**		

AutoRespTM software	Loligo Systems	N/A
Image J	National Institutes of Health, Bethesda, MD, United States	N/A
Xcalibur software	Thermo Fisher Scientific	N/A
Freestyle v.1.6	Thermo Fisher Scientific	N/A
MaxQuant MaxLFQ algorithm	Cox et al.^91^	N/A
Perseus software package (version 1.6.15.0)	Tyanova et al.^92^	N/A
Ilastik machine learning-based segmentation tool	Berg et al.^93^	N/A
Custom ImageJ script kindly provided by Professor György Hajnóczky (Thomas Jefferson University, Philadelphia, PA, USA)	Weaver et al.^94^	N/A
Zen software 2012 version	Zeiss AG, Oberkochen, Germany	N/A
Prism9	GraphPad, La Jolla, USA	N/A

**Other**		

Seahorse XFe96, respirometer	Agilent, Santa Clara, United States	N/A
Zebracube, Spontaneous locomotion tracking video system	Viewpoint, Lyon, France	N/A
170 mL swim tunnel respirometer	Loligo Systems, Viborg, Denmark	N/A
Spinning disk confocal microscope Nikon Ti2, CrEST Optics aX-Light V3	Nikon, Tokyo, Japan	N/A
20X Nikon objective - CFI Plan Apochromat Lambda	Nikon, Tokyo, Japan	N/A
Fusion Tribrid Orbitrap mass spectrometer	Thermo Fisher Scientific	N/A
Ultimate 3000 RSLCnano HPLC system	Dionex, Sunnyvale, United States	N/A
35 mm Dish, No. 0 Uncoated Coverslip, 14 mm Glass Diameter	MatTek, Ashland, MA, USA	P35G-0-14-C
Zeiss LSM510 laser scanning confocal microscope	Zeiss AG, Oberkochen, Germany	N/A
Zeiss 63X NA 1.4 oil immersion objective	Zeiss AG, Oberkochen, Germany	N/A
Transmission electron microscope Philips CM100	ThermoFisher Scientific	N/A
TVIPS TemCam-F416 digital camera	TVIPS GmbH, Gauting, Germany	N/A
Confocal microscope system Zeiss LSM 780	Zeiss AG, Oberkochen, Germany	N/A
Live imaging with a X40 oil immersion lens	Zeiss AG, Oberkochen, Germany	N/A

### Resource availability

#### Lead contact

Further information and requests for resources and reagents should be directed to and will be fulfilled by the lead contact, Francesca Amati (francesca.amati@unil.ch).

#### Materials availability

The Zebrafish line bcl2l13-/- generated in this study has been deposited in the ZFIN.org network (line designation: ula2).

#### Data and code availability

• The accession number for the publicly available proteomics dataset generated for this work is listed in the key resources table.
• No codes were used in this manuscript.
• Any additional information required to reanalyse the data reported in this paper is available from the lead contact upon request.

### Experimental model and study participant details

#### Zebrafish husbandry and strains

Zebrafish (Danio rerio, Oregon AB) were housed in ZebTEC racks (Techniplast, Varese, Italy) at the zebrafish facility of the Faculty of Biology and Medicine, maintained at 28°C and on a 14:10h light:dark cycle. All experiments in adult animals were performed on males between 12 and 13 months old, unless otherwise specified. Fish were fed twice a day with dry food (Gemma micro 300, Skretting, Tooele, UT, United States).

#### Ethical approval

Animal experimentation (licence number VD3391) and husbandry (licence number VD-H21) were approved by the Service de la consommation et des affaires vétérinaires (SCAV) of the Canton of Vaud.

### Method details

#### Generation of CRISPR/Cas9 knockout zebrafish

The guide RNA (gRNA) targeting *bcl2l13* transcript (ENSDART00000090156.5) was designed on https://chopchop.cbu.uib.no. The target sequence was selected in exon 2 (Figure S1). The gRNA (GGTCTTGATCTTGATGGCGAGGG) was produced and purified with Gene Art precision gRNA synthesis kit (Invitrogen, ThermoFischer Scientific, United States). gRNA and recombinant Cas9 nuclease (Invitrogen) were mixed at respectively 100 ng/μL and 0.5 ng/μL in a 200 mM solution of KCl. 1 nL of the mix was injected into one-cell stage AB embryos. F1 heterozygous animals were identified after fin clipping and PCR amplification of a *bcl2l13* fragment (Table S1). The sequence modification was visualized on Bis-acrylamide gels. The exact nature of the CRISPR/Cas9-induced mutation was confirmed by Sanger sequencing on fish from the F2 generation. Fish exhibiting a deletion of 5 nucleotides at the end of exon 2 were used as KO animals. Gene and protein nomenclature follow *The Genetic Nomenclature Guide* (Table S5).

#### Protein sequence alignment

FASTA files of amino acid sequences of human BCL2L13 and zebrafish Bcl2l13 were obtained from the public NCBI databases and aligned using Clustal Omega.

#### *In vivo* maximal oxygen consumption and swimming performance

Incremental swimming tests were performed in a 170 mL swim tunnel respirometer (Loligo Systems, Viborg, Denmark). The swim tunnel was submerged in a 20 L tank with housing water maintained at 28°C. An air pump was used to regulate oxygen concentration in the water. Oxygen consumption rate (MO_2_) was measured with AutoRespTM software (Loligo Systems). To determine initial value, the water from housing system was defined as 100% oxygen saturation while a Na_2_So_3_ solution was used to define 0% saturation. Tests were individually performed on zebrafish and started with 10 min acclimation with a flow rate of 10 cm/s. Then a stepwise increment in water velocity (2.5 cm/s every 5 min loop) was applied until the zebrafish stopped swimming. Maximal oxygen uptake (MO_2_max) was defined as the highest oxygen consumption rate obtained. Critical swimming (Ucrit) was calculated using the formula: Ucrit = Uf + US x (Tf/TS), where Uf (cm/s) is the highest velocity, US is the velocity increment, Tf (min) the time elapsed at fatigue velocity, TS (min) the prescribed interval time (manufacturer’s protocol). Fish weight and length were measured for each fish after completion of the incremental swimming test.

#### Spontaneous locomotion

Spontaneous locomotion was assessed with the tracking video system Zebracube (Viewpoint, Lyon, France). Fish were placed individually in Techniplast 0.7 L breeding tanks (9x15 cm) in 500 mL of fresh housing water. Six tanks were positioned under the camera system of the apparatus. To avoid socialization, opaque cardboards were placed between the tanks. After a 10 min acclimation in the dark, video recording was started for 10 min with light. Velocity thresholds were set up as following: pausing < 2 cm/s, slow velocity > 2 cm/s; Fast velocity > 7 cm/s.

#### Fast and slow muscle fibers cross sectional area

Skeletal muscle from 6 *bcl2l13* KO and 6 wild type (WT) fish were immediately collected after euthanasia. Muscles were placed in Optimal cutting temperature compound (OCT) (Thermo Fisher Scientific, Waltham, United States) and cryoprotected in cooled isopentane (Sigma-Aldrich, Saint-Louis, United States) for 30 minutes, before being placed in liquid nitrogen. Samples were stored at -80°C until further use. Skeletal muscles were sectioned at 20 μm in a cryostat (CM3050S, Leica Microsystems, Wetzlar, Germany) set at -22°C for chamber and object temperature. Sections were placed on precleaned glass slides (Thermo Fisher Scientific), fixed in 4% paraformaldehyde (PFA)(Carl Roth GmbH, Karlsruhe, Germany) for one hour, permeabilized in 0.5% Triton X-100 (Sigma-Aldrich) for 1.5 hour and blocked in 2% PBS-BSA (phosphate-buffered saline - bovine serum albumin) (Sigma-Aldrich) for one hour. Between each step, the slides were washed thrice in PBS (Dr. G. Bichsel AG, Interlaken, Switzerland). The sections were incubated with anti-myosin heavy chain antibodies (S58, Developmental Studies Hybridoma Bank, University of Iowa, Iowa City, IA, United States) diluted at 1:50 in PBS-BSA 2% overnight at 4°C. The samples were washed four times and incubated in goat anti-mouse IgG-FITC secondary antibodies (SC-2010, Santa Cruz Biotechnology, Dallas, TX, United States) diluted at 1:500 and phalloidin (SC-363797, Santa Cruz Biotechnology) at 1:5000 for 4 hours at room temperature (RT) in the dark and washed four times. The sections were incubated in Hoechst (H3570, Invitrogen Molecular Probes, Eugene, United States) diluted at 1:8000 for 2 minutes and washed four times. Slides were mounted with Mowiol (Sigma-Aldrich) covered with a 24x50 mm coverglass (Merck, Darmstadt, Germany).

Images were acquired using a spinning disk confocal microscope (Nikon Ti2, CrEST Optics aX-Light V3, Nikon, Tokyo, Japan) at a 20X magnification with a Nikon objective (CFI Plan Apochromat Lambda 20X, N.A. 0.75, W.D. 1.0 mm, spring-loaded) in tiles to observe whole muscles.

Image analyses were performed blinded. Six regions of interest (ROI) of 810x810 μm were used in fast muscle fibers and 4 ROI of 450x450 μm were used in slow muscle fibers. Muscle fibers cross sectional area (CSA) was measured using Fiji’s analyze particle’s function.^95^ All whole fibers present in each ROI were counted, partial or damaged fibers were excluded.

Computations for each fiber type were 1) Mean CSA per ROI = (SumofCSAinROIn)/(NumberoffibersinROIn) and 2) Mean CSA per fish = (SumofallmeanCSAperROI)/(NumberofROIs).

#### Electron microscopy qualitative ultrastructure analyses of zebrafish skeletal muscle

Electron microscopy was performed at the Electron Microscopy Facility of the Faculty of Biology and Medicine as previously described.^89^ Zebrafish skeletal muscle were dissected immediately after euthanasia. Cross-sections of 0.5 mm were cut with a razor blade and placed in PB 0.1 M / 2.5% glutaraldehyde / PFA 4% solution and fixed for 1 hour. The samples were washed three times in PB buffer and postfixed by a fresh mixture of osmium tetroxide 1% (EMS, Hatfield, PA, US) with 1.5% potassium ferrocyanide (Sigma, St Louis, MO, US) in PB buffer during 1 hour at RT. The samples were then washed three times 5 minutes in distilled water and dehydrated in acetone solution (Sigma) at graded concentration (30% - 40 min; 70% - 40 min; 100% 2 x 60 min). This was followed by infiltration in Epon resin (Sigma) at graded concentrations (Epon 1/3 acetone - 2 h; Epon 3/1 acetone – 2 h, Epon 4 h; Epon 12 h). The samples were then carefully positioned using binoculars in a flat position on an Aclar film (EMS, Hatfield, PA, US) inside a Gene Frame, (15 x 16 mm x 270 μm – 65 μl) (ThermoFischer Scientific) in 65 μl of Epon resin and finally polymerized for 48 h at 60°C in the oven.

Transverse ultrathin sections of 50 nm were cut on a Leica Ultracut (Leica Mikrosysteme GmbH, Vienna, Austria) and picked up on a copper slot grid 2 x 1 mm (EMS, Hatfield, PA, US) coated with a PEI film (Sigma). Sections were poststained with uranyl acetate (Sigma) 4% in H2O during 10 min, rinsed several times with H_2_O followed by Reynolds lead citrate in H_2_O (Sigma) during 10 min and rinsed several times with H_2_O.

Micrographs were taken with a transmission electron microscope Philips CM100 (ThermoFisher Scientificat an acceleration voltage of 80 kV with a TVIPS TemCam - F416 digital camera (TVIPS GmbH, Gauting, Germany). Two montages (190 x 190 μm) per fish were acquired in the fast fiber region in 8 KO and 8 WT animals. Large montage alignment were performed using Blendmont command-line program from the IMOD software.^96^ Analyses were performed blinded using ImageJ^97^ (National Institutes of Health, Bethesda, MD, United States).

#### Skeletal muscle contractile protein removal for mass spectrometry

Procedure adapted from Potts *et* al.^98^ Frozen zebrafish skeletal muscles were homogenized using a FastPrep-24 tissue homogenizer (MP Biomedicals, Santa Ana, CA, United States) with Lysing Matrix M, 2 mL tube ceramide beads (MP Biomedical) for 60 sec (4.0 rpm) in lysis buffer containing 40 mm Tris (pH 7.5) (Biosolve Chimie, Dieuze, France), 1 mM EDTA (Sigma-Aldrich), 5 mM EGTA (Sigma-Aldrich), 0.5% Triton X-100 (Sigma-Aldrich) and cOmplete™ Protease Inhibitor Cocktail (Roche, Basel, Switzerland). Homogenates were centrifuged 5 min at 2000 g. The pellet fractions containing enriched contractile fiber proteins and supernatant containing reduced fiber fraction were separated. Both fractions were cleaned by repeating the procedure 3 additional times. The supernatant fraction was diluted 1:10 in MeOH and proteins were precipitated overnight at -20°C for mass spectrometric analysis.

#### Proteomics analyses

##### Sample preparation and protein digestion

Samples were digested following a modified version of the iST method^99^ (named miST method). Based on tryptophane fluorescence quantification,^100^ 70-110 μg of proteins were resuspended at 2 μg/μl in miST lysis buffer (1% Sodium deoxycholate, 100 mM Tris pH 8.6, 10 mM DTT) and heated 5 min at 95°C then diluted 1:1 (v:v) with water. Reduced disulfides were alkylated by adding ¼ vol. of 160 mM chloroacetamide (32 mM final) and incubated for 45 min at RT in the dark. Samples were adjusted to 3 mM EDTA and digested with 1.0 μg Trypsin/LysC mix (Promega #V5073) for 1 h at 37°C, followed by a second 1 h digestion with an additional 0.5 ug of proteases. To remove sodium deoxycholate, two sample volumes of isopropanol containing 1% TFA were added to the digests, and the samples were desalted on a strong cation exchange (SCX) plate (Oasis MCX; Waters Corp., Milford, MA) by centrifugation. After washing with isopropanol/1%TFA, peptides were eluted in 200 μl of 80% MeCN, 19% water, 1% (v/v) ammonia, and dried by centrifugal evaporation.

##### Liquid Chromatography-Mass Spectrometry analyses, Fusion (low resolution MS/MS)

Data-dependent LC-MS/MS analyses of samples were carried out on a Fusion Tribrid Orbitrap mass spectrometer (Thermo Fisher Scientific) connected through a nano-electrospray ion source to an Ultimate 3000 RSLCnano HPLC system (Dionex, Sunnyvale, United States), via a FAIMS interface. Peptides (1 ug) were separated on a reversed-phase custom packed 45 cm C18 column (75 μm ID, 100Å, Reprosil Pur 1.9 um particles, Dr. Maisch, Ammerbuch, Germany) with a 4-90% acetonitrile gradient in 0.1% formic acid (total time 140 min). The MS acquisition method cycled through three compensation voltages (CV) (-40, -50, -60V) to acquire full MS survey scans at 120'000 resolution at each CV. A data-dependent acquisition method controlled by Xcalibur software (Thermo Fisher Scientific) optimized the number of precursors selected (“top speed”) of charge 2+ to 5+ from each survey scan, while maintaining a fixed scan cycle of 1.0 s per FAIMS CV. Peptides were fragmented by higher energy collision dissociation (HCD) with a normalized energy of 32%. The precursor isolation window was 1.6 Th, and the MS2 scans were done in the ion trap. The *m/z* of fragmented precursors was then dynamically excluded from selection during 60 s.

##### Data processing

Raw data files were separated in traces specific for each compensation voltage (CV) using Freestyle v.1.6 (Thermo Fisher Scientific). Data files were analysed with MaxQuant 1.6.14.0^101^ incorporating the Andromeda search engine.^102^ Cysteine carbamidomethylation was selected as fixed modification while methionine oxidation and protein N-terminal acetylation were specified as variable modifications. The sequence databases used for searching were the zebrafish (*Danio rerio*) reference proteome based on the UniProt database (www.uniprot.org, version of June 26^th^, 2020, containing 46’847 sequences), and a “contaminant” database containing the most usual environmental contaminants and enzymes used for digestion (keratins, trypsin, etc). Mass tolerance was 4.5 ppm on precursors (after recalibration) and 20 ppm on MS/MS fragments. Both peptide and protein identifications were filtered at 1% FDR relative to hits against a decoy database built by reversing protein sequences. Traces for different CVs were defined as fractions in MaxQuant and the “match between runs” option was activated between neighboring fractions. Quantitation was performed by the MaxLFQ algorithm^91^ to generate LFQ values.

##### Data analysis

All subsequent analyses were done with the Perseus software package (version 1.6.15.0).^92^ Contaminant proteins were removed, and normalized LFQ values were log2-transformed. Only proteins identified with at least 2 razor or unique peptides were retained and the table was filtered further to keep 4’495 proteins having at least 5/8 valid quantitative values in at least one of the two groups (WT/KO). After imputation of missing values (based on normal distribution using Perseus default parameters), a T-test was used to compare conditions, with permutation-based FDR correction for multiple testing (Q-value threshold <0.05). The difference of means obtained from the tests were used for 1D enrichment analysis on associated GO/KEGG annotations as described.^40^ The enrichment analysis was also FDR-filtered (Benjamini-Hochberg, Q-val<0.02).

##### Data availability

All raw MS data together with raw output tables are available via the ProteomeXchange^86^^,^^87^ data repository (www.proteomexchange.org) with the dataset identifier PXD044765.

#### RNA extraction and RT-qPCR

Using TRI reagent (Sigma-Aldrich), RNA isolation was performed following the manufacturer’s guidelines. In brief, zebrafish skeletal muscle samples were lysed using a mechanical pestle (Kimble Chase, Vineland, USA) in TRI Reagent (Sigma-Aldrich). RNA was isolated with chloroform (Sigma-Aldrich), precipitated with isopropanol (Sigma-Aldrich) and resuspended. Final RNA concentration was measured using an ND-1000 Spectrophotometer (Thermo Fisher Scientific).

One μg RNA was retrotranscribed using GoScript™ Reaction Buffer (Random Primers) and GoScript™ Enzyme Mix (Promega, Madison, USA). RT products were mixed with Power SYBR Green PCR Master Mix (Thermo Fisher Scientific) and with 600 nM of forward and reverse primers (Table S1). All primers were validated before use. Samples were analyzed with ViiA 7 Real-Time PCR System (Thermo Fisher Scientific). Relative expression of mRNA was estimated using the 2−ΔΔCT method using *ef1α* as housekeeping gene.

#### Mitochondrial area in fish skeletal muscle

Skeletal muscle from 6 bcl2l13 KO and 6 wild type (WT) 27 months old fish were immediately collected after euthanasia. Muscle sections were stained with Anti-MCTO1 (1:1000, ab14705, Abcam, Waltham, MA, United States), goat anti-mouse IgG-FITC secondary antibodies (SC-2010, Santa Cruz Biotechnology) diluted at 1:500 and phalloidin (SC-363797, Santa Cruz Biotechnology) at 1:5000, using the same protocol as for the fast and slow fibers staining. The sections were counter stained with Hoechst (H3570, Invitrogen Molecular Probes, Eugene, United States). Slides were mounted with Mowiol (Sigma-Aldrich).

Mitochondrial surface area was measured in 6 ROI of 203 X 203 μm, normalized to phalloidin surface area and an average of the 6 ROI was computed for each fish. Computations for mitochondrial are were $\sum_{ROI\;6}^{ROI\;1}\;\frac{MTCO1\;area}{Phalloidin\;area\;}/6$.

#### Mitochondrial morphology in fish skeletal muscle

Mitochondria morphology was evaluated in 6 bcl2l13 KO and 5 WT, one WT was removed due to poor image quality. Muscle transversal sections were processed as described above for electron microscopy. One image per fish (184 x 185 μm) was analyzed in a blind manner. Each image contained at least 5 fibers. In each fiber, one ROI was drawn in the cytosol (20 x 20 μm) and one ROI in the subsarcolemmal region (10 x 10 μm) to account for a sufficient number of mitochondria. Thus, a total of 5 ROIs in each region were analyzed per fish. Mitochondria were drawn along the outer membrane. Partial mitochondria were not included. Once all mitochondria were circled, overlays were saved and processed using the automated particle analyses from Fiji. Mitochondria number is the average number of mitochondria per fiber. Surface area is the mean area of all mitochondria circled. Perimeter is the length of the circle drawn. Feret and minFeret are maximum and minimum widths. Taken together these outcomes allow to obtain a comprehensive quantitative assessment of mitochondria morphology. Computation for mitochondrial morphology markers were $\sum_{ROI\;5}^{ROI\;1}\;Mitochondrial\;morphology\;parameters/5$.

#### Respirometry in zebrafish frozen skeletal muscle

Respirometry in frozen muscles from KO and WT zebrafish were measured with a Seahorse XFe96 respirometer (Agilent, Santa Clara, United States) as described in detail previously.^103^ In brief, frozen muscle samples were thawed in ice-cold PBS, minced, and homogenized in MAS (70 mM sucrose (Sigma-Aldrich), 220 mM mannitol (Sigma-Aldrich), 5 mM KH2PO4 (Sigma-Aldrich), 5 mM MgCl2 (Sigma-Aldrich), 1 mM EGTA (Sigma-Aldrich), 2 mM HEPES pH 7.4 (Sigma-Aldrich)) to which collagenase Type IV (1 mg/ml final concentration) (GIBCO, Thermo Fischer Scientific) was added after mincing. Homogenization was performed mechanically with 10 strokes in glass–glass Dounce homogenizer (Wheaton Glass tissue grinder, Fisher Scientific). Homogenates were centrifuged at 1000 g for 10 min at 4°C; then, the supernatant was collected to assess protein concentration. 40 μg of homogenates from each sample were loaded in triplicates into Seahorse XFe96 microplate in 85 μL of MAS. The loaded plate was centrifuged at 2000 g for 5 min at 4°C (without brake), and an additional 65 μL of MAS containing cytochrome c (10 μg/ml) (Sigma-Aldrich) was added to each well. Differential port injections of beta-nicotinamide adenine dinucleotide (NADH) (1 mM) (Sigma-Aldrich) or succinate (Succ) (Sigma-Aldrich) + rotenone (ROT) (5 mM + 2 μM) (Sigma-Aldrich), rotenone + antimycin A (AA) (2 μM + 4 μM) (Sigma-Aldrich), N,N,N',N'-Tetramethyl-1,4-phenylendiamin (TMPD) (Sigma-Aldrich) + ascorbic acid (Asc) (0.5 mM + 1 mM) (Sigma-Aldrich) and azide (50 mM) (Sigma-Aldrich) allowed to measure the activity of mitochondrial Complex I, Complex II and Complex IV respectively. These experiments were done at 28°C. Values were normalized to mitochondrial content measured with MitoTracker-Deep RED FM (M22426, Thermo Fisher Scientific) using a Cytation 3 plate reader (BioTek, Agilent).

#### Western-blot and protein quantification

Western blots were performed as previously described.^23^ 10-20 μg of protein from each muscle sample were loaded in SDS-Page gels. Transfer was performed on methanol-activated PVDF membrane (Bio-Rad Laboratories, Hercules, CA, United States) in tris-glycine (Biosolve Chimie) 20% ethanol buffer. After blocking, primary antibodies were incubated overnight at 4°C: mouse anti-MYH2 (1:500, A4.74, Santa Cruz Biotechnology), mouse anti-ATP5a (1:500, ab14748, Abcam, Waltham, MA, United States), mouse anti-PDHα (1:1000, ab110333, Abcam), rabbit anti-PDH E1α phospho-serine 293 (1:1000, ab177461, Abcam), rabbit anti-BCL2L13 (1:500, N-ter, HPA050377, Sigma), rabbit anti-BCL2L13 (1:500, center, HPA030994, Sigma), mouse anti-FACL4 (1:500, ab155282, Abcam), mouse anti-NDUFS3 (1:1000, ab14711, Abcam), mouse anti-SERCA2 (1:500, ab150435, Abcam), rabbit anti-RyR1 antibody (Marks’ lab, Columbia University, NY, USA, Cat. #: 5,029, Aa 1327–1339), rabbit anti-MCU (1:1000, HPA016480, Sigma), mouse anti-SERCA1 (1:1000, ab2819, Abcam), mouse anti-αTubulin (1:2000, T6199, Sigma), mouse anti-IP3R-I/II/III (1:200, sc-377518, Santa Cruz biotechnology) and rabbit anti-cleaved Caspase 3 (1:500, 9661S, Cell signaling, Danvers, United States). HRP-linked secondary antibodies anti-mouse IgG (1:5000, 7076, Cell signaling) and anti-rabbit IgG (1:5000, 7074, Cell signaling) were incubated for 2 h. Signal acquisition was performed on Biorad Chemidoc (ChemiDoc XRS+, Bio-Rad Laboratories) and quantified by densitometry using ImageJ^97^ (National Institutes of Health). Protein levels were normalized on the overall concentration corresponding to MemCode™ staining (Thermo Fisher Scientific) or αTubulin.

#### Cell cultures

Three types of cells were used for the specific experiments detailed in subsequent sections (Table S4). These included HeLa cells, human immortalized myoblasts (HIM) and C2C12. HeLa cells were grown in DMEM GlutaMAX (31966–021, GIBCO) supplemented with 10% fetal bovine serum (FBS) (10270–016, GIBCO) and 100 IU/ml penicillin (15-140-122, GIBCO), 100 μg/ml streptomycin (15140122, GIBCO). HIM were grown in DMEM low glucose (10567014, GIBCO) supplemented with 10% FBS (GIBCO), 10 ng/mL hEGF (PHG0311, GIBCO), 0.39 μg/mL Dexamethasone (D8893, Sigma-Aldrich), 0.5 mg/mL BSA (A8412, Sigma-Aldrich) and 0.5 mg/mL Fetuin (F3385, Sigma-Aldrich). C2C12 mouse skeletal myoblasts were grown in DMEM GlutaMAX (31966–021, GIBCO) supplemented with 10% FBS, 100 IU/ml penicillin, 100 μg/ml streptomycin (15140122, GIBCO) and maintained at 37°C in a humidified atmosphere with 5% CO_2_. To induce differentiation, myoblasts were grown to 80–90% confluence, proliferation medium was replaced with differentiation medium, consisting of DMEM low glucose (10567014, GIBCO) supplemented with 2% horse serum (26050088, GIBCO), 100 IU/ml penicillin and 100 μg/ml streptomycin. For specific experiments, C2C12 were cultured on 35 mm Dish, No. 0 Uncoated Coverslip, 14 mm Glass Diameter (MatTek, Ashland, MA, USA).

#### Subcellular fractioning and isolation of ERMCs

Adapting the protocol from Wieckowski et al.,^104^ HIM cells were trypsinized, centrifuged at 3x 600 g for 5 min in PBS and homogenized in isolation buffer 1 (225 mM mannitol, 75 mM sucrose, 0.1 mM EDTA, 30 mM Tris–HCl pH 7.4 (Sigma-Aldrich)) with 120 strokes at 2000 rpm with a glass–glass dounce homogenizer. All steps were performed at 4°C. After 3 subsequent centrifugation rounds at 600 g for 5 min, the supernatant was collected and centrifuged at 7000 g for 10 min. The supernatant contained ER and cytosol, and the pellet contained mitochondria and ERMCs. Further ER and cytosol were centrifuged at 20’000 g for 30 min, the supernatant was collected and further centrifuged at 100’000 g for 1 h. The pellet was ER and the supernatant was cytosol. For mitochondria and ERMCs, the pellet was resuspended in isolation buffer 2 (225 mM mannitol, 75 mM sucrose and 30 mM Tris–HCl pH 7.4) and centrifuged at 10’000 g for 10 min. The subsequent pellet was resuspended in mitochondria resuspending buffer (MRB) (250 mM mannitol, 5 mM HEPES pH 7.4 and 0.1 mM EDTA) using cut pipette tips. The resuspended pellet was layered on Percoll Medium (225 mM mannitol, 25 mM HEPES pH 7.4, 1mM EGTA and 30% Percoll vol/vol). The ultracentrifuge tubes were filled to the top with MRB buffer and centrifuged at 95’000 g for 30 min. The ring in the middle of the gradient was ERMCs, the pellet was mitochondria. ERMCs were collected and diluted 10x in MRB. The mitochondria pellet was resuspended in MRB. Both ERMCs and mitochondria were recentrifuged separately at 6300 g for 10 min. After centrifugation pure mitochondria pellet was collected for analysis. For ERMCs, the supernatant was centrifuged at 100’000 g for 1 h. The resulting pellet contained the ERMCs fraction.

#### HeLa SPLICS_S/L_-P2A-^ER-MT^ reporters

Split-GFP-based contact site sensors (SPLICS) were used in HeLa cells adapting the procedure described in Vallese *et* al.^90^ Using the plasmids SPLICS_S_-P2A^ER-MT^ and SPLICS_L_-P2A-^ER-MT^ (kindly provided by Professor Tito Calì, University of Padova, Padova, Italy), HeLa cells were transfected with Lipofectamine 3000 (L3000-01, Invitrogen) following supplier instructions. Confocal images were acquired at 405 and 488 nm excitation wavelength using a Zeiss LSM510 laser scanning confocal microscope (Zeiss AG, Oberkochen, Germany) and a 63X NA 1.4 oil immersion objective. A constant z-step of 0.32 μm was used to acquire z-stacks of the whole cellular volume. SPLICS signal was convolved and blurred using ImageJ functions. Stacks were processed using ImageJ VolumeJ plug-in.^56^ 3D rendering was segmented using Ilastik machine learning-based segmentation tool.^93^ Resulting binary mask was analyzed using ImageJ analyze particle function.

#### C2C12 myotubes *Bcl2l13* silencing

After 5 days of differentiation, myotubes were transfected with predesigned Mission esiRNA targeting mouse *Bcl2l13* (*siBcl2l13*, esiRNA1, Sigma-Aldrich) or with 30 nM Mission esiRNA targeting firefly luciferase (siControl, Sigma-Aldrich) using Lipofectamine RNAimax according to manufacturer’s instructions (Thermo Fisher Scientific). Myotubes were analyzed for all experiments 4 days post-transfection. For caspase-cleavage, thapsigargin 1 μM (T9033-5MG, Sigma-Aldrich) was added 3 days post-transfection for 18 hours.

#### ERMCs quantification in C2C12 knock down and control

Electron microscopy was performed at the Electron Microscopy Facility of the Faculty of Biology and Medicine. 27 micrographs of 4.95 x 4.95 μm per condition, siControl and *siBcl2l13* myotubes, were taken with a transmission electron microscope Philips CM100 (ThermoFisher Scientific) with a TVIPS TemCam-F416 digital camera (TVIPS GmbH, Gauting, Germany).

Image analyses were performed blinded using a custom ImageJ script kindly provided by provided by Professor György Hajnóczky (Thomas Jefferson University, Philadelphia, PA, USA).^94^ ERMCs were defined if the distance between ER and MOM was below 50 nm. ERMCs number per mitochondria, ERMCs length and ERMCs mean and minimal distances were quantified. Further outcomes included the number of mitochondria per cell and the perimeter of each mitochondria forming an ERMC.

#### Cytosolic Ca^2+^ imaging in C2C12

This procedure was performed in blinded fashion simultaneously in *siBcl2l13* and siControl myotubes. As previously described,^43^ cells were loaded with 5 μM of the cytosolic Ca^2+^ indicator Fluo-4 AM (Invitrogen) solubilized in a Ca^2+^ containing Krebs solution (in mM: NaCl 135.5, MgCl2 1.2, KCl 5.9, glucose 11.5, HEPES 11.5, CaCl2 1.8, final pH 7.3) for 20 min in the incubator. Cells were then rinsed twice with a Ca^2+-^free Krebs solution (in mM: NaCl 135.5, MgCl2 1.2, KCl 5.9, glucose 11.5, HEPES 11.5, 200 μM Na-EGTA, final pH 7.3). Fluo-4 fluorescence was monitored using a confocal microscope system Zeiss LSM 780 Live imaging with a X40 oil immersion lens. The excitation wavelength was 488 nm and the emitted fluorescence was recorded between 495 and 525 nm. After recording basal fluorescence, myotubes were stimulated with 2.5 mM caffeine (final concentration) (27602, Sigma) to trigger Ca^2+^ release from the SR. Zen software 2012 version (Zeiss) was used for the acquisition, and data were exported to excel files for analysis. The single excitation/emission Fluo-4 dye was normalized to pre-stimulation values to account for possible differences in dye loading and excitation strength. Unblinding took place after the complete analyses were finished.

#### Mitochondrial Ca^2+^ uptake in C2C12

This procedure was performed blinded simultaneously in *siBcl2l13* and siControl myotubes. As previously described,^43^ cells were loaded with 1ml of Krebs solution (in mM: NaCl 135.5, MgCl2 1.2, KCl 5.9, glucose 11.5, HEPES 11.5, CaCl2 1.8, final pH 7.3) containing 1 μM of the mitochondrial fluorescent indicator Rhod-2 AM (Invitrogen) for 1 hour at RT. Cells were then washed twice with Krebs solution and Rhod-2 fluorescence was measured using a confocal microscope Zeiss LSM 710 confocal microscope with a X40 oil immersion lens, with excitation at 532 nm and the emitted signal collected through a bandpass filter (540–625 nm). Change in Rhod-2 fluorescence was calculated by reporting the peak of fluorescence to the baseline (normalized fluorescence). Unblinding took place after the complete analyses were finished.

#### Gateway® cloning

For the plasmids Control-P2A-BFP, BCL2L13-P2A-BFP, Flag-*bcl2l13*-WT and KO, coding sequences were cloned from cDNA obtained from human muscle or zebrafish embryos at 48 hours post fertilization (hpf) and amplified by PCR with ATTB flanking sequences to use in the Gateway cloning system (Table S1). PCR products were purified on agarose gels (MinElute Gel Extraction kit, 28604, Qiagen, Hilden, Germany) and inserted in Gateways pDon221 vector by recombination using BP clonase II enzyme mix (11789, Invitrogen). For expression in eukaryote cells, CDS were transferred in destination vectors pcDNA-pCI-CMV-3XflagN^89^ or pCS Dest2 Attb1-3 (22424, Addgene, Watertown, MA, United States) + pDonR2R3-p2a-BFP with LR clonase II enzyme mix (11791, Invitrogen).

### Quantification and statistical analysis

After checking normality and equality of variance, two-tails independent *t*-tests were performed to examine group differences. If the equality of variance assumption was not met, comparisons between groups were performed with the Welch corrected *t*-test. If the normality assumption was not met, comparisons between groups were performed with the nonparametric Mann-Whitney test. All reported sample numbers refer to biological replicates unless otherwise indicated. A *P* value of <0.05 was considered statistically significant. All statistics and graphs were performed with Prism9 (GraphPad, La Jolla, USA).

## Supplemental information

Document S1. Figures S1–S6 and Tables S1 and S3–S5

Table S2. Proteomics sample table, related to Figure 3 and Table S3
