# Supplementary material for: BCL2L13 at endoplasmic reticulum-mitochondria contact sites regulates calcium homeostasis to maintain skeletal muscle function
Source: iScience. 2024 Jul 14;27(8):110510. doi: 10.1016/j.isci.2024.110510 (PMC11340602; doi:10.1016/j.isci.2024.110510)
Supplement: Document S1. Figures S1–S6 and Tables S1 and S3–S5 [file mmc1.pdf]

iScience, Volume *27*

Supplemental information

BCL2L13 at endoplasmic reticulum-mitochondria contact sites regulates calcium homeostasis to maintain skeletal muscle function

Dogan Grepper, Cassandra Tabasso, Nadège Zanou, Axel K.F. Aguettaz, Mauricio Castro-Sepulveda, Dorian V. Ziegler, Sylviane Lagarrigue, Yoan Arribat, Adrien Martinotti, Ammar Ebrahimi, Jean Daraspe, Lluis Fajas, and Francesca Amati

SUPPLEMENTAL INFORMATION

Table of content

Figure S1. Validation of the Bcl2l13 knockout model, related to STAR methods.

Figure S2. Zebrafish skeletal muscle proteomics, related to Figure 3.

Figure S3. Intermyofibrillar and subsarcolemmal mitochondria morphology in fast skeletal muscle fibers, related to Figure 4.

Figure S4. Key players in mitophagy, mitochondrial dynamics and Ca2+ uptake, related to Figure 3.

Figure S5. Silencing validation in C2C12 myotubes and expression of Ca2+ signalling proteins, related to Figures 5 and 6.

Figure S6. ERMCs in siBCL2L13 and siControl C2C12 myotubes, related to Figures 5.

Table S1. CRISPR genotyping primers, Gateway primers, QPCR primer sequences of target genes, related to STAR Methods.

Table S2. Proteomics sample table, related to Figure 3 (separate Excel file, not included in this supplemental package)

Table S3. Proteomics abbreviation list, related to Figure 3.

Table S4. Recapitulation of all models used in this manuscript, related to STAR Methods.

Table S5. Gene and protein nomenclature rules, related to STAR Methods.

Figure S1. Validation of the *Bcl2l13* knockout model, related to STAR Methods. (A) Amino acid sequence alignment of human *BCL2L13* (UniProt: Q9BXK5) and zebrafish *bcl2l13* (UniProt: Q1LX53). (B) Schematic representation of the CRISPR/Cas9 design. (C) Representative image of sequenced PCR products of WT and KO. (D) Western blot of HeLa cells expressing Flag-tagged *bcl2l13* cloned from WT (Flag-WT) or KO 48 hpf embryos (Flag-KO). (E) QPCR analysis from whole skeletal muscle of WT and KO fish. n=8 fish per group. Error bars are mean ± SEM, $^{**}P < 0.01$ (unpaired *t*-test). WT: Wild type and KO *bcl2l13*.

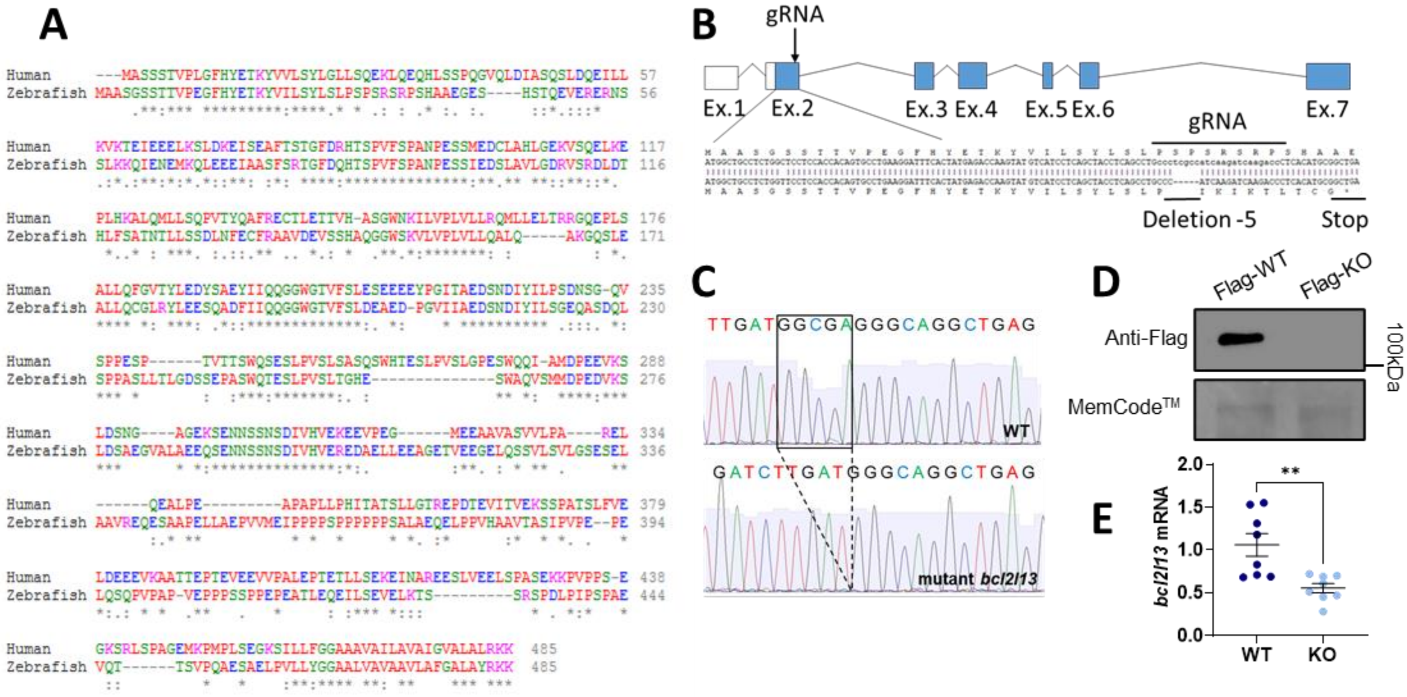

Figure S2. Zebrafish skeletal muscle proteomics, related to Figure 3. Enrichment score KO/WT of significantly modified protein groups related to (A) Gene Ontology Biological Process, (B) Gene Ontology Cellular Compartment. For all panels, n=8 fish per group.

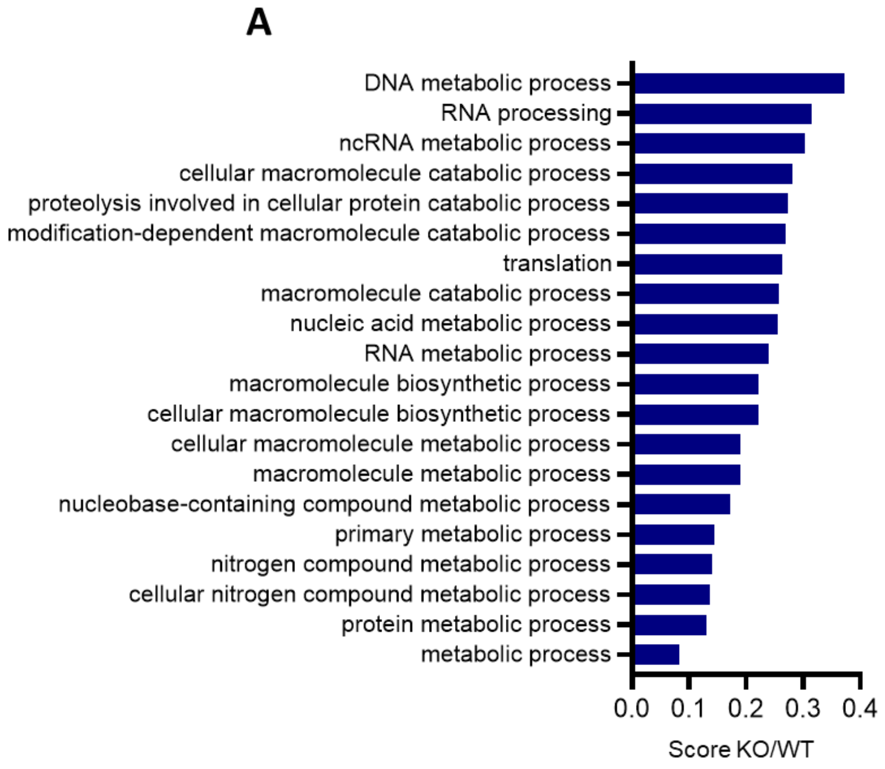

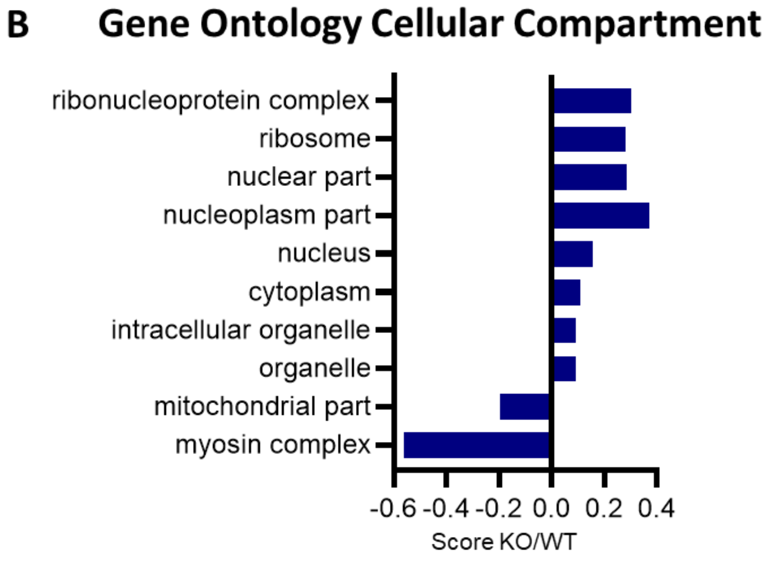

Figure S3. Intermyofibrillar and subsarcolemmal mitochondria morphology in fast skeletal muscle fibers, related to Figure 4. (A-M) Analyses of mitochondrial parameters on electron micrographs. n= 5 WT and 6 *bcl2l13* KO zebrafish. Error bars are mean ± SEM, **P* < 0.05, ***P* < 0.01 (unpaired t-test). WT: Wild type and KO: Mutant *bcl2l13*. IMF is intermyofibrillar, SS is subsarcolemmal, min Feret is minimum Feret, AR is aspect ratio.

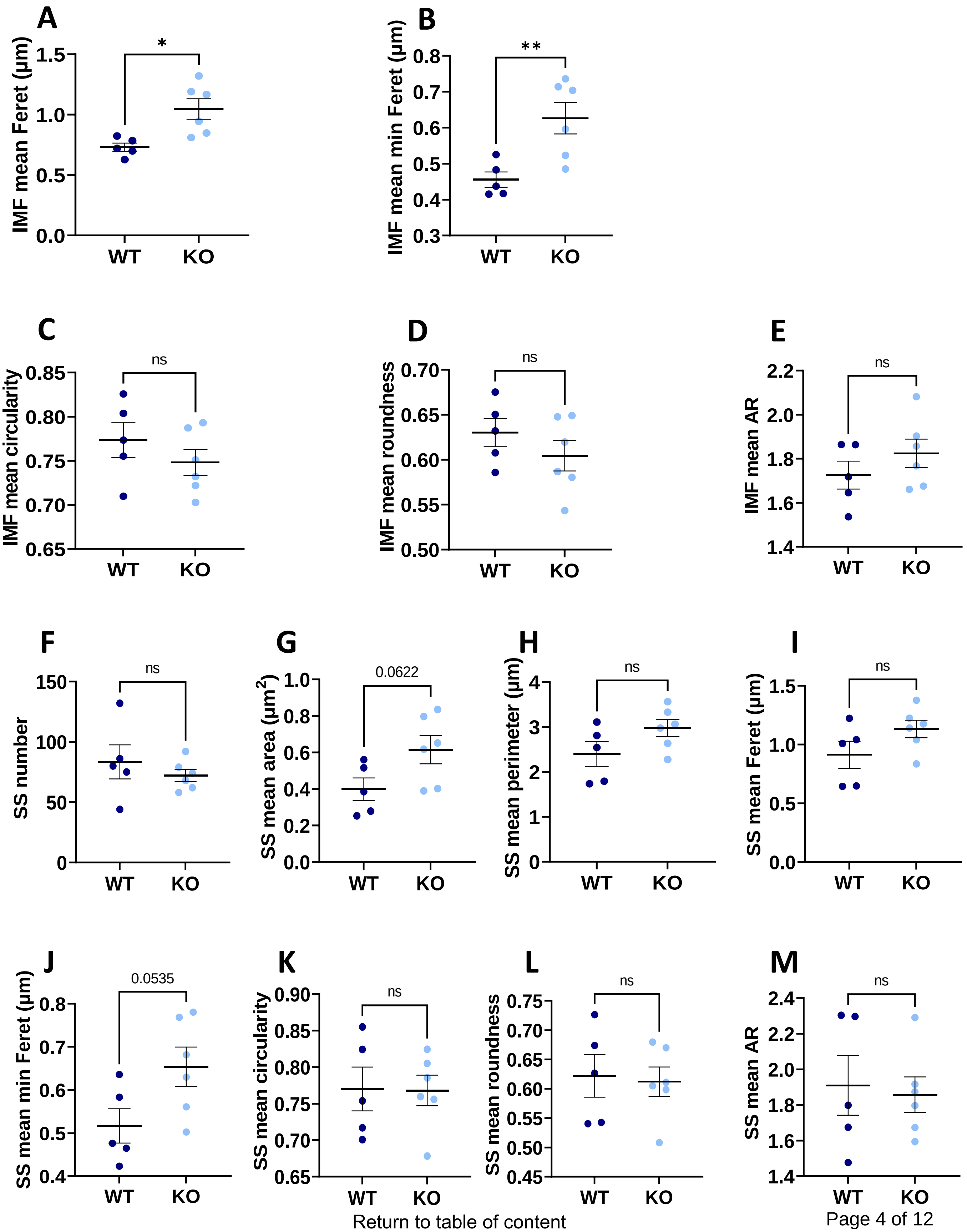

Return to table of content

Figure S4. Key players in mitophagy, mitochondrial dynamics and Ca^{2+} uptake, related to Figure 3. (A-J) qPCR analysis from whole skeletal muscle of WT and KO fish. (K) Representative western blot of skeletal muscle lysate after contractile protein removal, (L) Ratio of detected pPDH and tPDH normalized to total protein content (MemCode^{TM}). For all panels, n=8 biological replicates per group. Error bars are mean ± SEM, **P* < 0.05, ***P* < 0.01 (unpaired t-test).

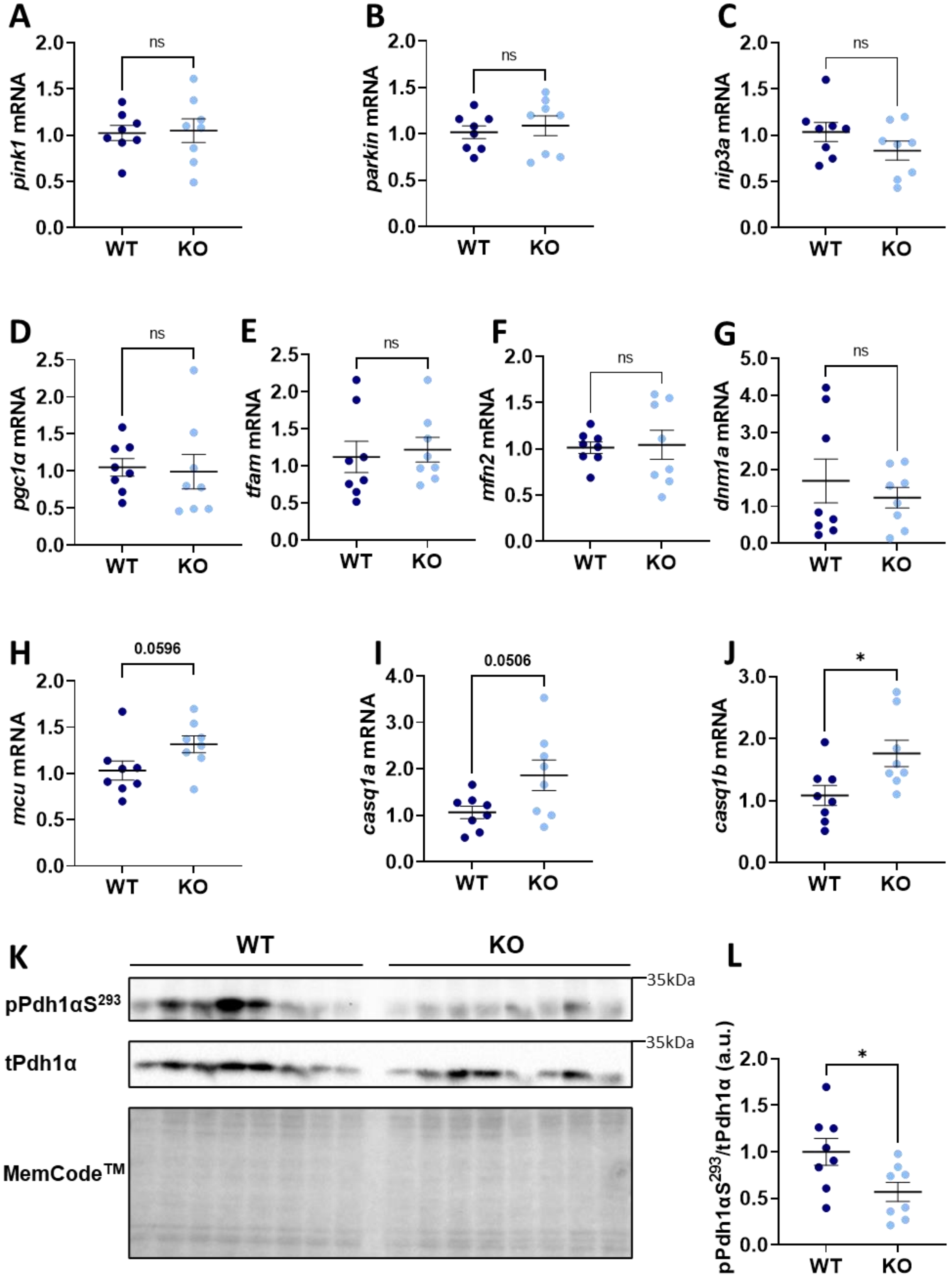

Figure S5. Silencing in C2C12 myotubes and expression of Ca^{2+} signalling proteins, related to Figures 5 and 6. (A) Representative western blot in mature C2C12 myotubes transfected with siControl or *siBCL2L13*. (B-E) Quantification of protein content detected by western blot in mature C2C12 myotubes transfected with siControl or *siBCL2L13* normalized to α-Tubulin or (F) ratio of pPDH1αS^{293}/tPDH1α normalized to α-Tubulin. (G) Representative western blot from mature C2C12 myotubes transfected with siControl or *siBCL2L13* in basal condition and after 18h of thapsigargin (TG) treatment. (H) Quantification of cleaved CASP3 detected by western blot of siControl and *siBcl2l13* myotubes treated for 18h with TG normalized to MemCode™. For all panels, n=4 independent transfections per condition. Error bars are mean ± SEM, *P < 0.05, ** P < 0.01 (unpaired t-test).

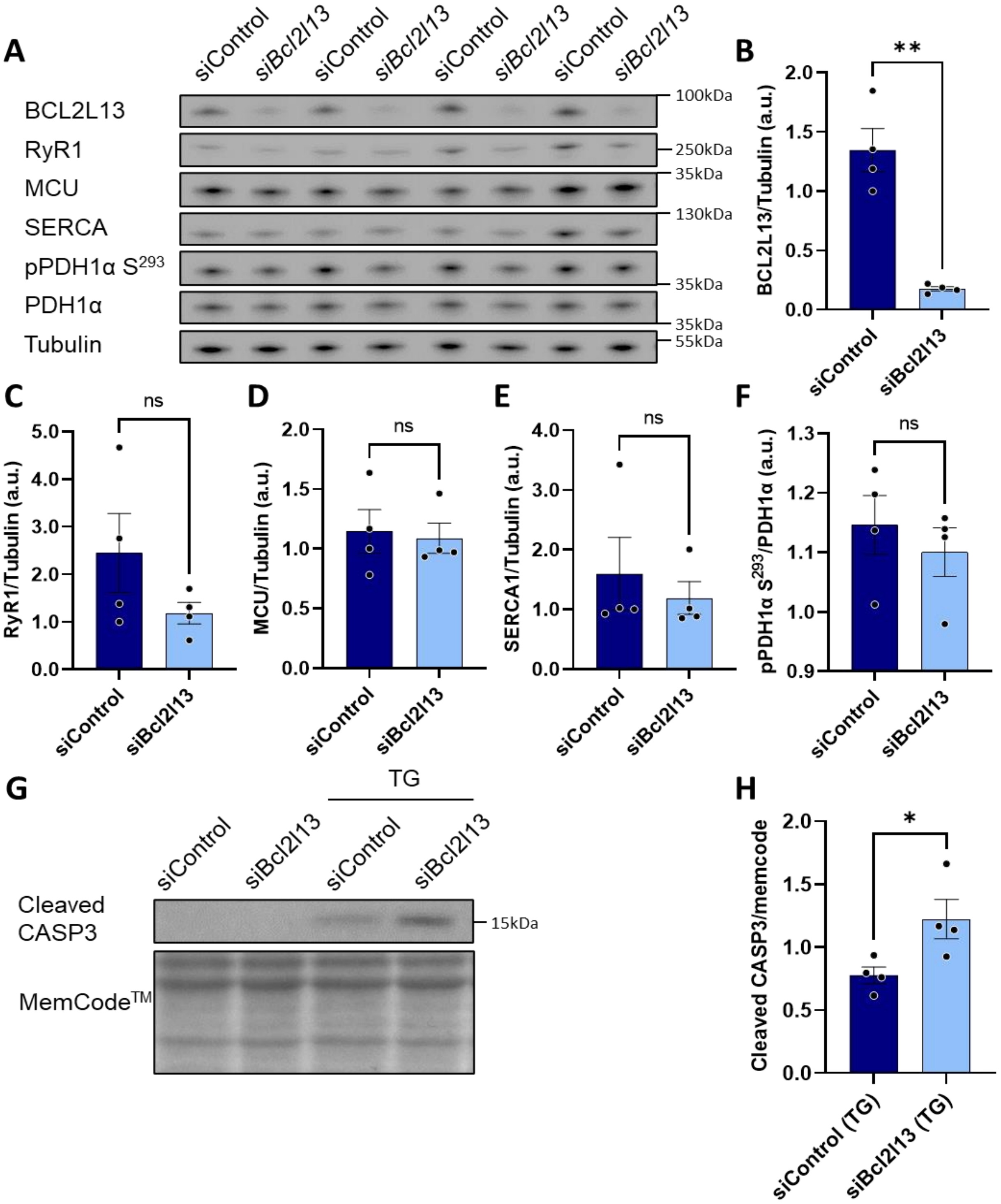

Figure S6. ERMCs in *siBCL2L13* and siControl C2C12 myotubes, related to Figure 5. (A) Number of ERMCs per cell. (B) Number of mitochondria per cell. (C) ER-mitochondria minimal distance. (D) ER-mitochondria mean distance. (D) Total mitochondria length in contact with ER. All parameters were measured in electron micrographs from siControl and *siBCL2L13* transfected C2C12 myotubes. n=27 micrographs (4.95x4.95µm) per condition from four independent experiments. For all panels, the median is shown by a white full line while quartiles are dotted lines, $^{\#}$ $P < 0.05$ (Mann-Whitney test).

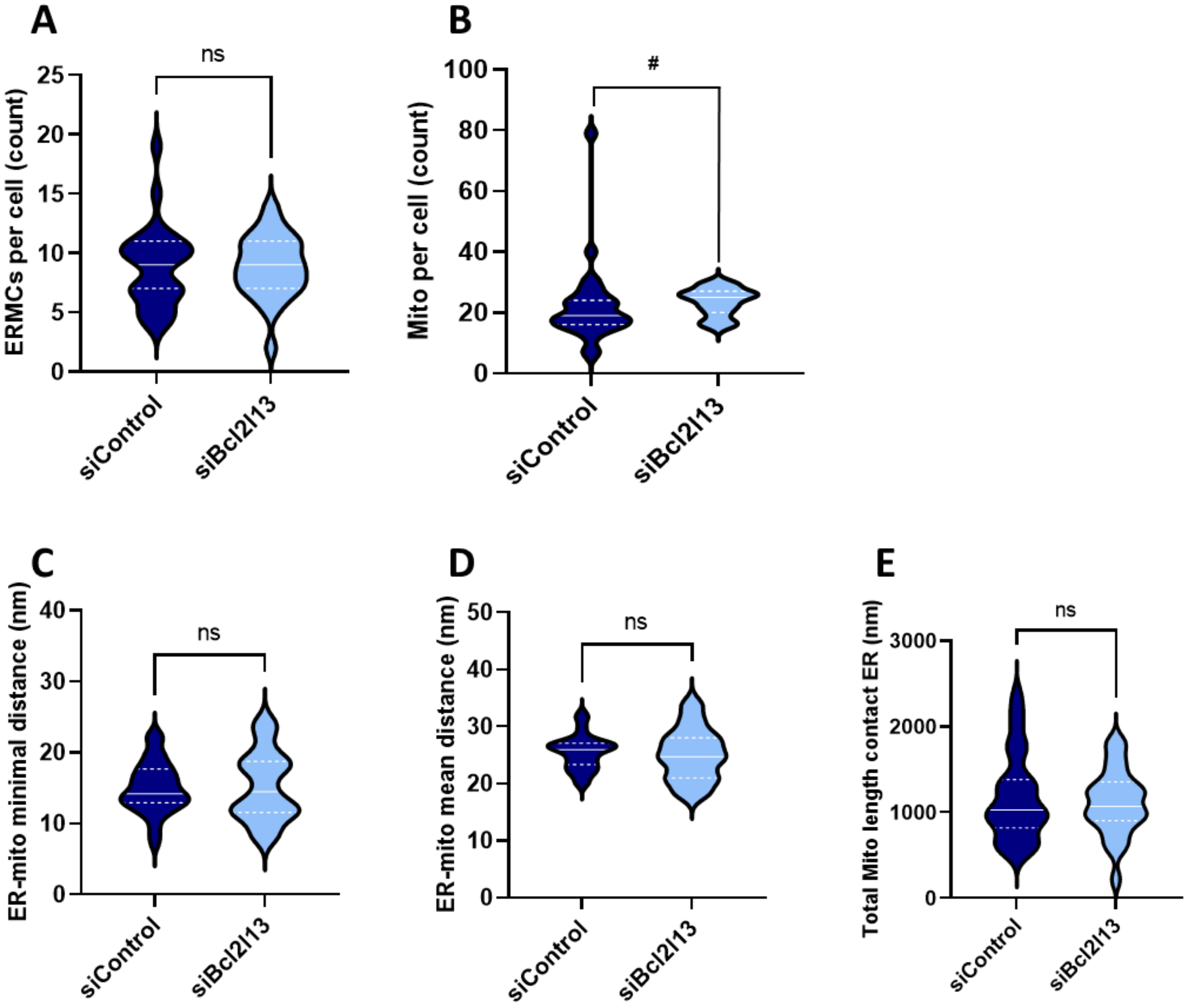

Table S1. CRISPR genotyping primers, Gateway primers, QPCR primer sequences of target genes, related to STAR Methods.

Method	Gene	Species	Name	sequence 5'-3'
CRISPR/Cas9	*bcl2l13*	zebrafish	CRISPR Genotyping For	GGTGTTAACTTTGTTTGTTTGCT
			CRISPR Genotyping Rev	CCTGACGATTTAACATTATTTCC
Gateway® Cloning	*bcl2l13*	zebrafish	ATTB1-*bcl2l13* -for	ggggacaagtttgtacaaaaaagcaggcttcAtggctgcctctggctcct
			ATTB2-*bcl2l13* -rev	ggggaccactttgtacaagaaagctgggttCttcttcctgtaggctagagc
	BCL2L13	human	ATTB1 *BCL2L13*- for	ggggacaagtttgtacaaaaaagcaggcttcATGGCGTCCTCTTCTACTGT
			ATTB2 *BCL2L13* -rev	ggggaccactttgtacaagaaagctgggttTTTCTTTCTCAGAGCCAGGGCTAC
	P2A-BFP		ATTB2_*P2A_BFP* -for	ggggacaagtttgtacaaaaaagcaggcttcGGAAGCGGAGCTACTAACTT
			ATTB3_*P2A_BFP* -rev	ggggaccactttgtacaagaaagctgggttATTAAGCTTGTGCCCCAGTTT
	UQCRC2		ATTB1-MLS	ggggacaagtttgtacaaaaaagcaggcttcATGTCCGTCCTGACGCCGCT
			ATTB2-MLS	ggggaccactttgtacaagaaagctgggttCCCCAACGAATGGATCTTGGC
QPCR	*casq1a*	zebrafish	*casq1a* _for	TGGGAAACCCTACAGTGAGAAAG
			casq1a _rev	GTCAAGAGCATCCTCCCAGATC
	casq1b		*casq1b* _for	CACGACAGACCGACTCTGAGAA
			casq1b _rev	ACTCCTCAGCAAAGGCAACAA
	mcu		*mcu* _for	CCTCATGGCAGGTTCAAAGAC
			mcu _rev	GACGGCAGACGTACAGAAATCA
	ef1α		*ef1α* _for	CCCCTGGACACAGAGACTTCATC
			ef1α _rev	ATACCAGCCTCAAACTCACCGAC

Table S3. Proteomics abbreviation list, related to Figure 3.

Abbreviation	Full name	Uniprot ID	
camk2a	**calcium/calmodulin-dependent protein kinas**	Q32PV2	Calcium signaling
casq1b	**Calsequestrin**	Q08BX6	
cacna1sa	**Voltage-dependent L-type calcium channel subunit alpha**	A0A8M2BFL7	
casq1a	**Calsequestrin**	A0A0R4IKE0	
capn3b	**Calpain-3**	E9QE31	
ryr1a	**Ryanodine receptor 1 isoform X5**	A0A8M9PLS9	
stac3	**SH3 and cysteine-rich domain-containing protein 3**	Q6DBR6	
stim1a	**Stromal interaction molecule 1 precursor**	Q1LY88	
jph2	**Junctophilin**	A4QNV2	
vdac1	**Voltage-dependent anion-selective channel protein 1**	Q6NWC1	
cacna2d1a	**Voltage-dependent calcium channel subunit alpha-2/delta-1 isoform X2**	A0A8M3ARM	
calm1a	**Calmodulin**	Q6PI52	
ryr1b	**Ryanodine receptor 1**	A6P4B8	
ryr3	**Ryanodine receptor 3 isoform X3**	A0A8M3ATC3	
cacna1sb	**Voltage-dependent L-type calcium channel subunit alpha**	Q6RKB0	
canx	**Calnexin**	F1QKS4	
sri	**Sorcin**	F1R4I7	
calr	**Calreticulin**	Q6PE26	
saraf	**Store-operated calcium entry-associated regulatory factor**	F1QPC3	
calr3b	**Calreticulin**	A0A0R4IL29	
micu2	**Calcium uptake protein 2, mitochondrial**	Q1LYC5	
mcu	**Calcium uniporter protein, mitochondrial**	Q08BI9	
micu1	**Calcium uptake protein 1, mitochondrial**	A4IG32	
sirt5	**NAD-dependent protein deacylase sirtuin-5, mitochondrial**	Q6DHI5	ROS
cat	**Catalase**	Q9PT92	
sod1	**Superoxide dismutase [Cu-Zn]**	O73872	
park7	**Parkinson disease protein 7 homolog**	Q5XJ36	
gpx4a	**Glutathione peroxidase**	A0A8M1PAF3	
slc25a24	**Mitochondrial adenyl nucleotide antiporter SLC25A24**	Q66L49	
gpx4b	**Glutathione peroxidase**	B5DDU3	
sirt2	**NAD-dependent protein deacetylase sirtuin-2**	Q7ZVK3	
txn2	**Thioredoxin 2**	Q6P131	
gpx3	**Glutathione peroxidase**	A0A8M1NIR5	
erp44	**Endoplasmic reticulum protein 44**	Q6P3G9	
sod3a	**Extracellular superoxide dismutase [Cu-Zn]**	A5PLA4	
gpx1a	**Glutathione peroxidase**	Q5XJ4	
txn	**Thioredoxin**	Q6DGI6	
acmsd	**2-amino-3-carboxymuconate-6-semialdehyde decarboxylase**	A4QP55	NAD+ metabolism
sirt5	**NAD-dependent protein deacylase sirtuin-5, mitochondrial**	Q6DHI5	
gpd1a	**Glycerol-3-phosphate dehydrogenase [NAD(+)]**	Q567A1	
nmnat3	**Nicotinamide-nucleotide adenylyltransferase**	B0V3M5	
slc25a51b	**Solute carrier family 25 member 51-like**	E7F6T6	
aldh2.1	**Aldehyde dehydrogenase, mitochondrial**	Q7SXU3	
afmid	**Kynurenine formamidase**	Q566U4	
aldh4a1	**Delta-1-pyrroline-5-carboxylate dehydrogenase, mitochondrial**	Q7SY23	

nampta	**Nicotinamide phosphoribosyltransferase**	E7F8T6	
nadk2	**NAD kinase 2, mitochondrial**	A0A8M2B8E7	
hibadhb	**3-hydroxyisobutyrate dehydrogenase**	Q7SXJ	
nnt	**proton-translocating NAD(P)(+) transhydrogenase**	Q6NYQ7	
gpd1l	**Glycerol-3-phosphate dehydrogenase 1-like protein**	Q8N335	
gpd1c	**Glycerol-3-phosphate dehydrogenase [NAD(+)]**	Q7T3H5	
aldh5a1	**Succinate-semialdehyde dehydrogenase**	A0A0R4IIB7	
gpd2	**Glycerol-3-phosphate dehydrogenase, mitochondrial**	A0A8M2BDB8	
gpd1b	**Glycerol-3-phosphate dehydrogenase [NAD(+)]**	Q7T1E0	
coa6	**Cytochrome c oxidase assembly factor 6**	B8A5J2	
cox6b1	**Cytochrome c oxidase subunit**	Q6DH63	
cox4i1l	**Cytochrome c oxidase subunit 4**	F1R6I1	
cox8b	**Cytochrome c oxidase subunit 8B**	X1WER9	
cox7a3	**Cox7a2l protein**	Q7SXI1	
cox7a1	**Cytochrome c oxidase subunit 7A1, mitochondrial**	Q08CE7	
cox6a2	**Cytochrome c oxidase subunit**	Q66I77	
cox5aa	**Cytochrome c oxidase subunit 5A, mitochondrial**	Q4VBU7	
cox6c	**Cytochrome c oxidase subunit 6C**	Q6ZM23	
cox4i1	**Cytochrome c oxidase subunit 4**	Q6TNV0	CIV activity
cox7c	**Cytochrome c oxidase subunit 7C, mitochondrial**	Q6PBP0	
cox6b2	**Cytochrome c oxidase subunit**	Q7SXM1	
cox5b2	**Cytochrome c oxidase subunit 5B2**	Q0P3U4	
cox7a2a	**Cytochrome c oxidase subunit 7A2, mitochondrial**	Q504B4	
mt-co2	Cytochrome c oxidase subunit 2	Q9MIY7	
mt-co1	**Cytochrome c oxidase subunit 1**	Q9MIY8	
cox7b	**Cytochrome c oxidase subunit 7B, mitochondrial**	B3DJA6	
surf1	**SURF1-like protein**	A0A8M3B2I7	
cox5ba	**Cytochrome c oxidase subunit 5B, mitochondrial**	Q6IQU9	

Table S4. Recapitulation of all models used in this manuscript, related to STAR Methods.

Model	Purpose	Rationale	Figure
**C2C12**	Ca^{2+} flux, ERMCs contact sites	Accurate model of muscle function, only contractile cellular model, accurate organelle architectures	5E-G, S5, S6, 6
**HIM**	BCL2L13 organelle specific localization	Human cells, accurate model of human organelle structures	5A
**HeLa**	SPLICS analysis for ERMCs contact distance	Transfectable, accurate image of organelle morphology	S1, 5B-D
**Zebrafish muscle**	Muscle function, fiber type analysis, mitochondrial dynamics, morphology and function, physiological role of BCL2L13	Follows the 3R principles, Accurate model of human muscle morphology, two spatially distinct fiber types, accurate model of human muscle organelle dynamics and morphology	1, 2, 3, S2, 4, S3, S4

Table S5. Gene and protein nomenclature, related to STAR Methods. Throughout the manuscript, nomenclature rules are adapted from *The Genetic Nomenclature Guide*, published in 1998 in *Trends in Genetics*, Elsevier.

Model	Gene	Protein
**C2C12 (mouse)**	Shh	SHH
**HIM, HeLa (human)**	SHH	SHH
**Zebrafish muscle**	*shha*	Shha
